# Abdominal and gluteo-femoral markers of adiposity and risk of vascular-metabolic mortality in a prospective study of 150 000 Mexican adults

**DOI:** 10.1093/eurjpc/zwab038

**Published:** 2021-03-09

**Authors:** Louisa Gnatiuc, Roberto Tapia-Conyer, Rachel Wade, Raúl Ramirez-Reyes, Diego Aguilar-Ramirez, William Herrington, Michael Hill, Sarah Lewington, Jason Torres, Eirini Trichia, Rory Collins, Richard Peto, Robert Clarke, Pablo Kuri-Morales, Jonathan R Emberson, Jesus Alegre-Díaz

**Affiliations:** Clinical Trial Service Unit and Epidemiological Studies Unit (CTSU), Nuffield Department of Population Health (NDPH), University of Oxford, Oxford, UK; School of Medicine, National Autonomous University of Mexico, Mexico City, Mexico; Clinical Trial Service Unit and Epidemiological Studies Unit (CTSU), Nuffield Department of Population Health (NDPH), University of Oxford, Oxford, UK; MRC Population Health Research Unit, NDPH, University of Oxford, Richard Doll Building, Old Road Campus, Oxford OX3 7LF, UK; School of Medicine, National Autonomous University of Mexico, Mexico City, Mexico; Clinical Trial Service Unit and Epidemiological Studies Unit (CTSU), Nuffield Department of Population Health (NDPH), University of Oxford, Oxford, UK; Clinical Trial Service Unit and Epidemiological Studies Unit (CTSU), Nuffield Department of Population Health (NDPH), University of Oxford, Oxford, UK; MRC Population Health Research Unit, NDPH, University of Oxford, Richard Doll Building, Old Road Campus, Oxford OX3 7LF, UK; Clinical Trial Service Unit and Epidemiological Studies Unit (CTSU), Nuffield Department of Population Health (NDPH), University of Oxford, Oxford, UK; MRC Population Health Research Unit, NDPH, University of Oxford, Richard Doll Building, Old Road Campus, Oxford OX3 7LF, UK; Clinical Trial Service Unit and Epidemiological Studies Unit (CTSU), Nuffield Department of Population Health (NDPH), University of Oxford, Oxford, UK; MRC Population Health Research Unit, NDPH, University of Oxford, Richard Doll Building, Old Road Campus, Oxford OX3 7LF, UK; UKM Medical Molecular Biology Institute (UMBI), Universiti Kebangsaan Malaysia, Kuala Lumpur, Malaysia; Clinical Trial Service Unit and Epidemiological Studies Unit (CTSU), Nuffield Department of Population Health (NDPH), University of Oxford, Oxford, UK; Clinical Trial Service Unit and Epidemiological Studies Unit (CTSU), Nuffield Department of Population Health (NDPH), University of Oxford, Oxford, UK; Clinical Trial Service Unit and Epidemiological Studies Unit (CTSU), Nuffield Department of Population Health (NDPH), University of Oxford, Oxford, UK; Clinical Trial Service Unit and Epidemiological Studies Unit (CTSU), Nuffield Department of Population Health (NDPH), University of Oxford, Oxford, UK; Clinical Trial Service Unit and Epidemiological Studies Unit (CTSU), Nuffield Department of Population Health (NDPH), University of Oxford, Oxford, UK; School of Medicine, National Autonomous University of Mexico, Mexico City, Mexico; Clinical Trial Service Unit and Epidemiological Studies Unit (CTSU), Nuffield Department of Population Health (NDPH), University of Oxford, Oxford, UK; MRC Population Health Research Unit, NDPH, University of Oxford, Richard Doll Building, Old Road Campus, Oxford OX3 7LF, UK; School of Medicine, National Autonomous University of Mexico, Mexico City, Mexico

**Keywords:** Abdominal adiposity, Gluteo-femoral adiposity, Cause-specific mortality, Mexico, Prospective cohort study

## Abstract

**Aims:**

Results of previous studies of abdominal adiposity and risk of vascular-metabolic mortality in Hispanic populations have been conflicting. We report results from a large prospective study of Mexican adults with high levels of abdominal adiposity.

**Methods and results:**

A total of 159 755 adults aged ≥35 years from Mexico City were enrolled in a prospective study and followed for 16 years. Cox regression, adjusted for confounders, yielded mortality rate ratios (RRs) associated with three markers of abdominal adiposity (waist circumference, waist–hip ratio, and waist–height ratio) and one marker of gluteo-femoral adiposity (hip circumference) for cause-specific mortality before age 75 years. To reduce reverse causality, deaths in the first 5 years of follow-up and participants with diabetes or other prior chronic disease were excluded. Among 113 163 participants without prior disease and aged 35–74 years at recruitment, all adiposity markers were positively associated with vascular-metabolic mortality. Comparing the top versus bottom tenth of the sex-specific distributions, the vascular-metabolic mortality RRs at ages 40–74 years were 2.32 [95% confidence interval (CI) 1.84–2.94] for waist circumference, 2.22 (1.71–2.88) for the waist–hip ratio, 2.63 (2.06–3.36) for the waist–height ratio, and 1.58 (1.29–1.93) for hip circumference. The RRs corresponding to each standard deviation (SD) higher usual levels of these adiposity markers were 1.34 (95% CI 1.27–1.41), 1.31 (1.23–1.39), 1.38 (1.31–1.45), and 1.18 (1.13–1.24), respectively. For the markers of abdominal adiposity, the RRs did not change much after further adjustment for other adiposity markers, but for hip circumference the association was reversed; given body mass index and waist circumference, the RR for vascular-metabolic mortality for each one SD higher usual hip circumference was 0.80 (0.75–0.86).

**Conclusions:**

In this study of Mexican adults, abdominal adiposity (and in particular the waist–height ratio) was strongly and positively associated with vascular-metabolic mortality. For a given amount of general and abdominal adiposity, however, higher hip circumference was associated with lower vascular-metabolic mortality.

## Introduction

Excess adiposity is a major global cause of chronic disease^1^ with overweight and obesity estimated to account for about 4 million deaths annually worldwide.^2^ Prospective studies conducted in European or North American populations have reported that abdominal adiposity is more hazardous than general adiposity [usually measured by body mass index (BMI)] for risk of death from vascular-metabolic causes.^3–6^ A Mendelian randomization study, involving 0.5 million European adults in the UK Biobank, reported strong positive associations of genetically instrumented waist–hip ratio with risks of premature death from coronary artery disease, ischaemic stroke, diabetes, liver disease, and renal failure (along with non-vascular and non-metabolic diseases), thereby providing support for the causal relevance of central adiposity for such diseases.^7^ However, the available evidence on the associations of central adiposity with cause-specific mortality in Latin-American populations is limited and conflicting, involving mostly adults aged over 60 years and focusing largely on all-cause mortality, with some studies reporting higher risks of death associated with markers of abdominal adiposity and others showing no such associations.^8–11^

Comparisons of previous observational studies have been complicated by use of different markers of central adiposity, different approaches for analysis of such markers (e.g. categorical or continuous), and varying approaches to limit the effects of reverse causality bias due to pre-existing disease. In a meta-analysis of 58 prospective studies conducted mainly in high-income countries, and involving 221 934 adults without prior history of major chronic diseases, each one standard deviation (SD) higher level of both waist circumference and waist–hip ratio were associated with about 25% higher risk of cardiovascular disease.^5^ Likewise, a large multi-centre European study reported similar findings,^6^ but previous studies of Hispanic populations living in Latin-America failed to demonstrate any excess risk of death associated with higher levels of abdominal adiposity.^8–10^ More recently, the NHANES study of 10 329 Mexicans living in USA reported weak positive associations of both waist circumference and waist–height ratio with risks of death from vascular or metabolic causes.^11^

We have previously reported that general and, particularly, abdominal adiposity were strongly associated with all-cause mortality in a contemporary prospective cohort of 150 000 adults from Mexico City.^12^ However, that report did not compare the relevance of different markers of abdominal adiposity to specific causes of death, or assess the role of gluteo-femoral adiposity. The aims of the present analyses were to compare the overall and independent relevance of different markers of abdominal vs. gluteo-femoral adiposity with risks of death from vascular-metabolic and non-vascular-metabolic causes in the Mexico City Prospective Study.

## Methods

### Recruitment and follow-up surveys

Between 1998 and 2004, 159 755 adults aged 35 years or older, residing in two districts of Mexico City, were invited to participate in a prospective cohort.^13^ Age, sex, socio-economic status, lifestyle factors, current medication, and medical history were recorded by trained staff. Weight, height, waist and hip circumferences were measured to the nearest 0.1 kg or 0.1 cm, respectively, using calibrated electronic scales, stadiometers and non-stretchable tapes. A 10 mL blood sample was collected from each participant and the plasma and buffy coat samples were sent to Oxford (UK) for long-term storage over liquid nitrogen. Glycosylated haemoglobin (HbA1c) was measured in buffy coat samples using a validated high-performance liquid chromatography method.^14^ Between 2015 and 2019, repeat measures of anthropometry (and other characteristics) were recorded in a random subset of 10 144 survivors. Ethics committee approval was obtained in both Mexico and the UK. All the participants provided written informed consent.

### Mortality follow-up

Mortality was tracked until January 2018 through probabilistic linkage to the Mexican Electronic Death Registry (*Sistema Epidemiológico y Estadístico de las Defunciones*), which encodes all diseases listed on the death certificate to the 10th Revision of the International Classification of Diseases (ICD-10).^15^ Field-validation of a subset of about 7000 matched deaths confirmed the reliability of the matching process in 95% of the cases. Study clinicians reviewed and, where necessary, recoded the underlying cause of death (e.g. accepting diabetes as the underlying cause only for acute diabetic crises).^16^ Vascular-metabolic deaths included vascular deaths (which were further categorized as cardiac, cerebrovascular, or other vascular) and metabolic deaths (including renal deaths, acute diabetic deaths and deaths from hepatobiliary disease) (Supplementary material online, *Webtable S1*). Other deaths (i.e. non-vascular-metabolic) were categorized as neoplastic, respiratory, infective, and other.

### Statistical analyses

To reduce the risk of reverse causality bias (in particular the effects of poorly controlled diabetes on weight prior to recruitment),^12^ the main analyses excluded participants with self-reported previously diagnosed diabetes, taking regular antidiabetic medication or with baseline glycosylated haemoglobin ≥6.5% (suggestive of screen-detected diabetes in the absence of a diagnosis), in addition to participants with a prior diagnosis of angina, myocardial infarction, stroke, cancer, chronic kidney disease, cirrhosis, or emphysema. Analyses were restricted to deaths occurring before age 75 years and further excluded all deaths during the first 5 years of follow-up (to minimize the risk of reverse causality). Thus, the prospective analyses shown are of deaths at ages 40–74 years.

We used Cox regression to assess the relevance of three markers of abdominal adiposity (waist circumference, waist–hip ratio, and waist–height ratio) and one marker of gluteo-femoral adiposity (hip circumference) to risk of death from particular causes at ages 40–74 years. (Some analyses of BMI were also done for comparative purposes.) For each adiposity marker, participants were first classified into baseline groups defined by the fifths of the sex-specific distributions. Then, in order to further explore the nature of any dose–response risk-relationships at the extremes of the distribution, we divided the top and bottom groups into two equal groups, thereby yielding a total of seven groups (i.e. the top and bottom two-tenths of each distribution plus the middle three-fifths). The Cox analyses relating these seven groups to mortality risk were adjusted for age-at-risk (in 5-year ranges), sex, district of residence (two districts), self-reported highest level of education attained (university or college, high school, elementary school, or other), leisure-time physical activity (none, up to twice weekly, at least three times weekly), tobacco use (never, former, occasional, <10 cigarettes per day, ≥10 cigarettes per day), and alcohol consumption (never, former, current). To compare the overall versus independent (i.e. mutually adjusted) relevance of each marker of adiposity to risk, these analyses were conducted before and after inclusion of other markers of adiposity in the models. Multicollinearity was considered by looking at the pairwise correlations between variables and examining the change in magnitude of the standard errors in the resulting models (inflated standard errors being suggestive of unstable coefficients). The proportional hazards assumption was assessed through examination of the Schoenfeld residuals (no such tests were significant).

Group-specific mortality rate ratios (RRs) and 95% confidence intervals (CIs) were estimated for each baseline-defined adiposity group (including for the reference group^17^ with a RR of 1.0). These RRs were then plotted against the expected mean ‘usual’ level in each group, calculated as *u = M + r[b − M]*, where *b* is the group-specific observed mean baseline level, *M* is the overall mean, and *r* is the regression dilution ratio,^18^ which was estimated to be 0.84 for waist circumference, 0.90 for hip circumference, 0.74 for waist–hip ratio, and 0.88 for waist–height ratio (see Supplementary material online, *Webfigure S1* for details). These estimates of *r* were also used to estimate the usual SD of each adiposity marker from the observed (i.e. baseline) SD through the equation s* = √r × *s*, where *s* was the average of the observed SD in men and the observed SD in women.^19^ The average mortality RR corresponding to a one SD higher usual level of each adiposity marker was then calculated by performing a weighted regression through the seven log RR estimates (with weights equal to the inverse of the variances of the log RRs) and rescaling the resulting slope (and its SE) to correspond to one usual SD.

Sensitivity analyses included estimates by strata of confounders (with tests of heterogeneity or trend across levels of each confounder), further adjustment for dietary factors and sleep, inclusion of those with undiagnosed diabetes, exclusion of current or former smokers, and analyses without adjustment for regression dilution bias. All analyses were performed using SAS v9.4, STATA v14.1 and R v3.6.1 (www.r-project.org).

## Results

### Study participants

Among 112 333 eligible households visited at recruitment, 106 059 (94%) yielded a total of 159 755 potential participants. Of these, 12 116 (8%) were excluded because they were aged 75 years or older at recruitment. In addition, a further 6714 (4%) were excluded because of a prior history of chronic disease (other than diabetes), and a further 24 902 (16%) were excluded because of a history of diabetes or blood levels of glycosylated haemoglobin ≥6.5% at baseline. Of the remaining 116 023 participants, 2860 (2%) were excluded because of missing or implausible data (see footnote of *Table 1*), leaving 113 163 participants aged 35–74 years for the main analysis.

**Table 1:** Characteristics of 113 163 participants aged 35–74 years at recruitment, by sex

	Men (36 646)	Women (76 517)	All (113 163)
Age (years)	50 (11)	49 (10)	49 (10)
Socio-economic status and lifestyle behaviours			
Resident of Coyoacán	16 497 (45%)	30 321 (40%)	46 818 (41%)
University/college educated	9978 (27%)	10 605 (14%)	20 583 (18%)
Current smoker	19 094 (52%)	19 247 (25%)	38 341 (34%)
Current drinker	31 066 (85%)	53 998 (71%)	85 064 (75%)
Any regular leisure-time physical activity	11 508 (31%)	14 710 (19%)	26 218 (23%)
Physical measurements			
Height (cm)	165 (7)	152 (6)	156 (9)
Weight (kg)	76 (12)	68 (12)	71 (13)
BMI (kg/m^2^)	28.0 (4.1)	29.5 (5.0)	29.1 (4.8)
Waist circumference (cm)	96 (10)	92 (12)	94 (11)
Hip circumference (cm)	101 (8)	106 (11)	105 (10)
Waist–hip ratio	0.95 (0.06)	0.87 (0.06)	0.90 (0.07)
Waist–height ratio	0.58 (0.06)	0.61 (0.08)	0.60 (0.08)
SBP (mmHg)	127 (15)	124 (16)	125 (15)
DBP (mmHg)	84 (10)	82 (10)	83 (10)
Glycosylated haemoglobin			
Mean (SD), %	5.4 (0.4)	5.5 (0.4)	5.5 (0.4)
Long-term medication use			
Any anti-hypertensive	2752 (8%)	9700 (13%)	12 452 (11%)
Any anti-thrombotic	730 (2%)	1939 (3%)	2669 (2%)
Any lipid lowering	149 (<0.5%)	314 (<0.5%)	463 (<0.5%)

BMI, body mass index; DBP, diastolic blood pressure; SBP, systolic blood pressure.

Mean (SD) or *n* (column %) shown. Table excludes participants with previously diagnosed diabetes, those without previously diagnosed diabetes but with a glycosylated haemoglobin concentration at recruitment of 6.5% or greater, those with chronic disease (ischaemic heart disease, stroke, chronic kidney disease, cirrhosis, cancer, or emphysema) at recruitment, those with missing data on any analysis covariate (sex, district of residence, educational level attained, smoking status, alcohol intake, leisure time physical activity), uncertain follow-up, or missing or extreme measures of anthropometry: height (cm) <120 or >200, weight (kg) <35 or >250, BMI (kg/m^2^) <18.5 or ≥60, waist circumference (cm) <60 or >180, hip-circumference (cm) <70 or >180, waist–hip ratio <0.5 or >1.5.

### Adiposity-related characteristics at baseline

Among the 113 163 participants (mean baseline age 49 years), mean height was 165 (SD 7) cm in men and 152 (SD 6) cm in women, mean BMI was 28.0 (SD 4.1) kg/m^2^ in men and 29.5 (SD 5.0) kg/m^2^ in women, and mean waist circumference was 96 (SD 10) cm in men and 92 (SD 12) cm in women (*Table 1*). Higher levels of abdominal adiposity were associated with lower levels of leisure-time physical activity, education and current smoking, higher levels of blood pressure and use of anti-hypertensive medication, and higher levels of HbA1c (Supplementary material online, *Webtables S2a*–*e* show these associations as well as associations with hip circumference and BMI). The correlations between waist circumference, hip circumference, BMI, waist–hip ratio, and waist–height ratio are shown in Supplementary material online, *Webtable S3*.

### Associations with vascular-metabolic mortality

**Figure 1:**
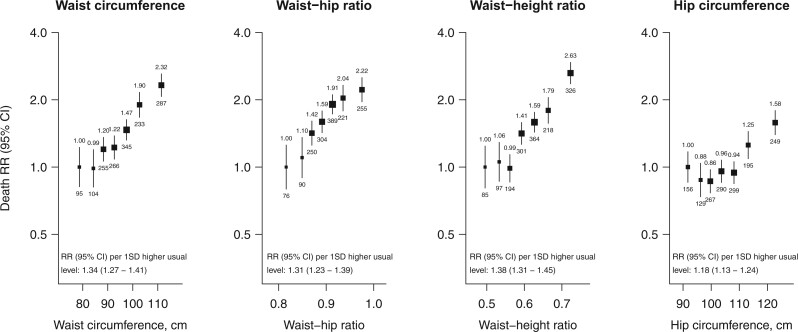
Adiposity and vascular-metabolic mortality at ages 40–74. Analyses exclude participants with an HbA1c level of ≥6.5% at recruitment, those with diabetes or other chronic diseases (ischaemic heart disease, stroke, chronic kidney disease, cirrhosis, cancer, or emphysema), and all deaths in the first 5 years of follow-up. The seven groups shown correspond to the top and bottom two-tenths and the middle three-fifths of each distribution. The RRs for the seven categories were plotted at the mean usual level in each group, with the vertical lines through each point representing group-specific 95% confidence intervals (with the area of each plotting symbol proportional to the amount of statistical information). RRs were adjusted for age-at-risk, sex, district of residence, self-reported highest level of education attained, leisure-time physical activity status, smoking status, and alcohol intake. The RR is noted above each vertical line and the number of deaths below. The average mortality RR per 1 SD higher usual level (9.9 cm for waist circumference, 8.8 cm for hip circumference, 0.052 for waist–hip ratio, 0.066 for waist–height ratio) is shown throughout the full range studied, and is calculated by performing a weighted regression through the seven log RR estimates (with weights equal to the inverse of the variances of the log RRs). For associations which do not appear to be log-linear, these average RRs may reflect different underlying RRs at different levels of adiposity.

At ages 40–74 years, each of the four markers of adiposity were positively associated with vascular-metabolic mortality, with approximately log-linear associations observed for waist circumference, waist–hip ratio, and waist–height ratio, and a curvilinear association observed for hip circumference (*Figure 1*). Comparing the top versus bottom tenth of each distribution, the vascular-metabolic mortality RR was 2.32 (95% CI 1.84–2.94) for waist circumference, 2.22 (1.71–2.88) for the waist–hip ratio, 2.63 (2.06–3.36) for the waist–height ratio, and 1.58 (1.29–1.93) for hip circumference. The RRs corresponding to one SD higher usual levels of these adiposity markers were 1.34 (95% CI 1.27–1.41), 1.31 (1.23–1.39), 1.38 (1.31–1.45), and 1.18 (1.13–1.24), respectively. The associations observed were broadly similar in subgroup analyses at different levels of age, sex or other confounders (Supplementary material online, *Webfigures S2* and *S3*).

*Table 2* shows the mortality RRs associated with one SD higher usual levels of each adiposity marker for the different types of vascular-metabolic death (see Supplementary material online, *Webfigures S4a*–*d* for the *shapes* of these associations). For each adiposity marker, the mortality RRs were broadly comparable for cardiac, cerebrovascular, other vascular, and hepatobiliary causes of death. For each adiposity marker, the most extreme associations were observed for deaths from renal disease and acute diabetic crises.

**Table 2:** Relevance of markers of adiposity to cause-specific vascular-metabolic mortality at ages 40–74 years, before and after mutual adjustment for other adiposity markers

Cause of death	No. of deaths	Death RR (95% CI) per 1 SD higher usual level^a^							
		Waist circumference		Waist–hip ratio		Waist–height ratio		Hip circumference	
		With basic adjustments	+hip-c +BMI	With basic adjustments	+BMI	With basic adjustments	+hip-c +weight	With basic adjustments	+waist-c +BMI
Vascular									
Cardiac	641	1.25 (1.15–1.35)	1.28 (1.14–1.44)	1.23 (1.11–1.35)	1.17 (1.05–1.29)	1.27 (1.18–1.38)	1.32 (1.20–1.46)	1.13 (1.05–1.22)	0.80 (0.72–0.89)
Stroke	215	1.29 (1.11–1.49)	1.45 (1.19–1.78)	1.29 (1.08–1.53)	1.23 (1.04–1.47)	1.43 (1.24–1.64)	1.77 (1.50–2.10)	1.11 (0.97–1.27)	0.78 (0.65–0.94)
Other vascular	84	1.48 (1.18–1.85)	1.54 (1.10–2.15)	1.32 (1.00–1.74)	1.26 (0.95–1.68)	1.52 (1.24–1.87)	1.44 (1.11–1.87)	1.38 (1.15–1.67)	1.16 (0.87–1.54)
Subtotal: any vascular	940	1.28 (1.19–1.37)	1.35 (1.22–1.48)	1.25 (1.15–1.35)	1.19 (1.09–1.29)	1.33 (1.25–1.42)	1.43 (1.32–1.54)	1.15 (1.08–1.22)	0.82 (0.75–0.90)
Metabolic									
Renal/acute diabetic crisis	256	1.60 (1.40–1.82)	1.57 (1.30–1.89)	1.38 (1.18–1.61)	1.22 (1.04–1.42)	1.60 (1.40–1.83)	1.56 (1.33–1.83)	1.39 (1.24–1.55)	0.85 (0.72–1.00)
Hepatobiliary	389	1.32 (1.19–1.47)	1.60 (1.38–1.86)	1.40 (1.24–1.59)	1.34 (1.18–1.52)	1.35 (1.22–1.49)	1.55 (1.37–1.75)	1.13 (1.03–1.24)	0.72 (0.63–0.82)
Subtotal: any metabolic	645	1.43 (1.31–1.55)	1.59 (1.41–1.79)	1.39 (1.27–1.54)	1.29 (1.17–1.42)	1.44 (1.33–1.56)	1.55 (1.41–1.71)	1.23 (1.15–1.33)	0.77 (0.70–0.86)
All vascular-metabolic	1585	1.34 (1.27–1.41)	1.44 (1.34–1.55)	1.31 (1.23–1.39)	1.23 (1.15–1.31)	1.38 (1.31–1.45)	1.48 (1.39–1.57)	1.18 (1.13–1.24)	0.80 (0.75–0.86)

RR estimates are adjusted for sex, age at risk, district of residence, educational level, smoking, alcohol intake, and leisure-time physical activity (basic adjustments). Analysis of each marker of adiposity with further mutual adjustment as described in the adjacent column header.

^a^ One standard deviation (SD) higher usual level of the adiposity markers was 9.9 cm for waist circumference, 0.052 for waist–hip ratio, 0.066 for waist–height ratio, and 8.8 cm for hip circumference.

### Mutual adjustment for other markers of adiposity

**Figure 2:**
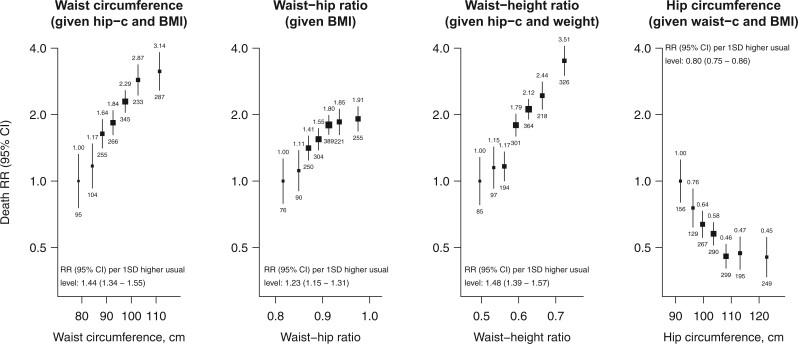
Adiposity and vascular-metabolic mortality at ages 40–74 after mutual-adjustment for other adiposity markers. Analyses, conventions and exclusions as for *Figure 1*. Independent relevance was assessed after mutual adjustment as follows: waist circumference given hip circumference and BMI, waist–hip ratio given BMI, waist–height ratio given hip circumference and weight, and hip circumference given waist circumference and BMI.

**Figure 3:**
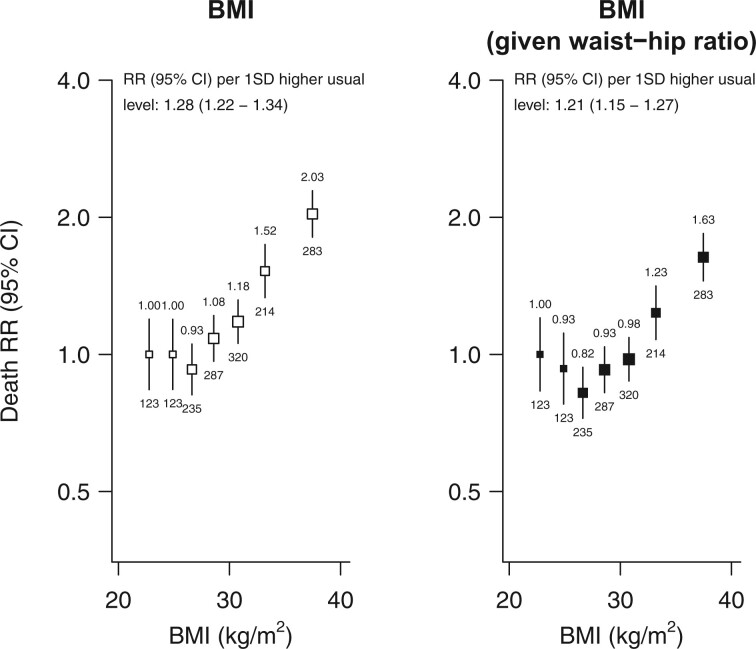
Overall and independent relevance of BMI to vascular-metabolic mortality at ages 40–74. Analyses, conventions and exclusions as for *Figures 1* and *2*. One SD usual BMI was 4.3 kg/m^2^. Independent relevance of BMI to vascular-metabolic mortality was assessed after mutual adjustment for waist-hip ratio. Among those with baseline BMI >25 kg/m^2^, the RR per 1 SD higher usual BMI was 1.36 (1.28–1.43) before mutual adjustment for WHR and 1.32 (1.25–1.40) after.

After adjustment for other markers of adiposity, the associations of waist circumference and of waist–height ratio with vascular-metabolic mortality were more extreme while the association of waist–hip ratio with vascular-metabolic mortality was attenuated (*Figure 2*). By contrast, the association of hip circumference with vascular-metabolic mortality was *reversed* when adjusting for waist circumference or BMI (and particularly when adjusting for both). (Despite some strong correlations between the adiposity markers (Supplementary material online, *Webtable S3*), the standard errors of the estimated log RRs in these adjusted models were not much different to those in the unadjusted models, indicating no major influence of collinearity.) The vascular-metabolic RRs comparing the top versus bottom tenth of the distribution, after adjustment for all other adiposity markers, were 3.14 (95% CI 2.17–4.54) for waist circumference, 1.91 (1.46–2.51) for waist–hip ratio, 3.51 (2.58–4.77) for waist–height ratio, and 0.45 (0.33–0.63) for hip circumference. For each 1 SD higher usual adiposity level, the mutually adjusted vascular-metabolic RR was 1.44 (95% CI 1.34–1.55) for waist circumference, 1.23 (1.15–1.31) for waist–hip ratio, 1.48 (1.39–1.57) for waist-height ratio, and 0.80 (0.75–0.86) for hip circumference. These mutually adjusted RRs were broadly similar at different levels of age, sex, or other confounders (Supplementary material online, *Webfigures S5* and *S6*). The shapes and strengths of the adiposity-adjusted associations for particular types of vascular-metabolic mortality are shown in *Table 2* and Supplementary material online, *Webfigures S7a*–*d*. (For comparison with the analyses of abdominal and gluteo-femoral adiposity, parallel analyses of BMI, both before and after adjustment for waist-hip ratio, are shown in *Figure 3*).

The RRs associated with each marker of adiposity (before and after mutual adjustment for other adiposity markers) were consistent when the analyses were further adjusted for dietary factors and sleep duration, included those with undiagnosed diabetes, or restricted to never-smokers (Supplementary material online, *Webtables S4a*–*c*). Supplementary material online, *Webtable S4d* also shows the associations before adjustment for regression dilution bias.

### Associations with non-vascular-metabolic mortality

Each of the four markers of adiposity were positively associated with higher risks of death from non-vascular-metabolic causes (Supplementary material online, *Webtable S5*). The non-vascular-metabolic mortality RRs corresponding to a one SD higher usual level were 1.18 (95% CI 1.12–1.23) for waist circumference, 1.16 (1.10–1.23) for waist–hip ratio, 1.18 (1.12–1.23) for waist–height ratio, and 1.11 (1.06–1.16) for hip circumference. After adjustment for other markers of adiposity, the associations of waist circumference with non-vascular-metabolic mortality were more extreme, but the associations of waist–hip and waist–height ratio with non-vascular-metabolic mortality were largely unaltered, and the associations of hip circumference with non-vascular-metabolic mortality disappeared (and slightly reversed). Among non-vascular-metabolic causes of death, associations were weaker for neoplastic mortality, but stronger for respiratory and infective causes of death (Supplementary material online, *Webtable S5* and *Webfigures S7a*–*d*).

## Discussion

In this Mexican population, abdominal adiposity was strongly and positively associated with risk of death from cardiac, cerebrovascular, renal, and other metabolic causes. Associations of waist circumference, waist–hip ratio, and waist–height ratio with vascular-metabolic mortality were broadly log-linear and largely independent of each other. Indeed, for waist circumference and waist–height ratio, the associations with vascular-metabolic mortality *increased* after adjustment for other markers of adiposity, such that individuals in the top tenth of each distribution had (for given levels of other adiposity markers) more than three times the risk of death from any vascular-metabolic cause compared with those in the bottom tenth of each distribution. The associations of these markers of abdominal adiposity with mortality were more extreme for deaths from renal disease and acute diabetic crisis. In contrast, the mortality associations with hip circumference (reflecting gluteo-femoral adiposity) with vascular-metabolic mortality were reversed after adjustment for waist circumference and BMI.

The findings of the present study of Mexican adults are consistent with those from European and other Western populations in the Emerging Risk Factors Collaboration (ERFC)^6^ and the European Prospective Investigation on Cancer and Nutrition (EPIC).^5^ However, the RRs observed for vascular mortality in the present study were more extreme than those reported in a previous study of Mexican Americans,^11^ and differ markedly from previous studies in Hispanic populations in which higher levels of abdominal adiposity were not associated with any excess risks of death from vascular and metabolic causes (possibly reflecting incomplete control for reverse causality bias in such studies).^8–11^ The RRs for vascular-metabolic mortality associated with markers of abdominal adiposity observed in the present study were weaker than those observed in Mendelian randomisation studies,^7^ perhaps because the latter approach estimates mortality risks associated the *life-time* effects of excess adiposity (including potentially additive effects of obesity in adolescence). Indeed, in a study of 2.3 million people aged 17 years at the time of measurement, the excess vascular mortality associated with higher levels of BMI were also more extreme than those estimated in the present study.^20^^,^^21^

The associations of abdominal adiposity with vascular-metabolic causes of death are likely to be causal. The mechanisms underlying such associations with deaths from vascular-metabolic causes include increased levels of blood pressure,^22^ impaired liver function, insulin resistance, glucose intolerance, inflammation and dyslipidaemia, elevated levels of non-esterified fatty-acids and pro-coagulation factors, and reduced clearance of apolipoprotein-B, triglycerides and very-low-density lipids.^23^^,^^24^ Given BMI and waist circumference, higher levels of hip circumference were strongly *inversely* associated with vascular-metabolic mortality. This is consistent with cross-sectional analyses of the EPIC-Norfolk study in which higher levels of hip circumference were associated with *lower* levels of total and low-density lipoprotein cholesterol (and with higher levels of high-density lipoprotein cholesterol) when adjusting for BMI and waist circumference,^25^ and supports the hypothesis that distribution of adiposity or body fat accumulation in the upper and lower parts of the body have opposing associations with risks of vascular-metabolic causes of death.^23^

This is the first large study to compare the independent relevance of several different markers of abdominal adiposity and of gluteo-femoral adiposity with mortality from vascular-metabolic causes in Mexican adults, and builds on previous findings for myocardial infarction from the INTERHEART study of a multi-ethnic population.^26^ In the present study, among the different markers of abdominal adiposity studied, the waist–height ratio was the most strongly associated with risk of death from vascular-metabolic causes, closely followed by waist circumference and the waist–hip ratio. Existing tools for predicting the 10-year risk of vascular disease recommend using BMI when cholesterol or other blood biomarkers are unavailable.^27^ Our findings suggest that inclusion of a marker of abdominal adiposity rather than, or perhaps in addition to, BMI, may further improve risk prediction. In contrast with previous recommendations by the 2008 World Health Organization Expert Consultation on waist circumference and waist–hip ratio,^28^ which highlighted the need for clinically informative adiposity cut-off points for Hispanic populations, the findings of the present study argue against the use of Mexican-specific cut-offs for markers of adiposity in clinical practice.

### Strengths and limitations

The chief strengths of the present study are the large number of participants studied and the prolonged duration of follow-up (resulting in a large number of deaths for analysis) together with careful control for reverse causality bias (by exclusion of individuals with previously diagnosed or undiagnosed diabetes, other diseases, and deaths occurring during the first 5 years of follow-up) in a previously-understudied population. In addition, the availability of standardized, directly measured anthropometry (rather than self-reports) and of repeat measurements in a subset of survivors, enabled us to estimate associations with long-term ‘usual’ (rather than baseline) levels of adiposity. Nevertheless, the present study had several limitations, including lack of imaging measures of abdominal adiposity or other potentially important ectopic depots (such as neck circumference),^29^ lack of data on blood levels of lipids, renal and liver function, which could have enabled us to explore the mechanisms underlying of associations of abdominal adiposity with mortality from various vascular or metabolic causes. Future Mendelian Randomization studies within the cohort will allow for assessments of the causal impact of lifelong differences in central adiposity. Additional limitations include the potential for residual confounding, a lack of information on non-fatal events (e.g. incident diabetes) and a reliance on the causes of death listed on the death certificate. However, almost all deaths in Mexico are certified by a doctor and the overall accuracy and quality of certification of causes of death in Mexico is high.^30^ Moreover, the validity of the attributed causes of death on the death certificates is supported by the specificity of the associations identified.

## Conclusions

In this Mexican population with a high prevalence of overweight and obesity, markers of abdominal adiposity were strongly and positively associated with risk of death from vascular, renal, and other metabolic causes, supporting the hypothesis that visceral adiposity is particularly hazardous to vascular and metabolic health. Given levels of other adiposity markers, the steeply *inverse* association of hip circumference with risk of death from these diseases suggests that fat preferentially stored around the hips (rather than viscerally) is protective for risks of death from vascular-metabolic causes.

## Supplementary material

Supplementary material is available at *European Journal of Preventive Cardiology* online.

## Data availability

We welcome requests from researchers who wish to access data from the Mexico City Prospective Study. If you are interested in obtaining data from the study for research purposes, or in collaborating with us on a specific research proposal, please visit our study website (https://www.ctsu.ox.ac.uk/research/prospective-blood-based-study-of-150-000-individuals-in-mexico) where you can download our Data and Sample Access Policy in either English or Spanish.

## Funding

This study was supported by grants from the Wellcome Trust, the Mexican Health Ministry, the National Council of Science and Technology for Mexico, Cancer Research UK, the British Heart Foundation, and the UK Medical Research Council Population Health Research Unit. D.A.-R. acknowledges support from the BHF Centre of Research Excellence, Oxford (grant code RE/13/1/30181).

**Conflict of interest:** R.C. holds a British Heart Foundation Chair, and reports personal fees from UK Biobank and grants from Merck & Co, the Medicines Company (now Novartis) and Pfizer, outside the submitted work. R.C. has a patent for a statin-related myopathy genetic test licensed to University of Oxford from Boston Heart Diagnostics (but has waived any personal reward). W.H. and J.R.E. report grants from Boehringer Ingelheim outside the submitted work, and W.H. reports a UK Medical Research Council (MRC)-Kidney Research UK Prof David Kerr Clinician Scientist Award. All other authors declare no competing interests.

## Supplementary Material

zwab038_Supplementary_Data
