# Supplementary material for: Abdominal and gluteo-femoral markers of adiposity and risk of vascular-metabolic mortality in a prospective study of 150 000 Mexican adults
Source: Eur J Prev Cardiol. 2021 Mar 9;29(5):730–8. doi: 10.1093/eurjpc/zwab038 (PMC9071366; doi:10.1093/eurjpc/zwab038)
Supplement: zwab038_Supplementary_Data [file zwab038_supplementary_data.pdf]

Supplementary material

Abdominal and gluteo-femoral markers of adiposity and risk of vascular-metabolic mortality in a prospective study of 150,000 Mexican adults

Figures

		Pg
1.	Extent of regression to the mean for particular markers of adiposity	2
2.	Age and sex-specific relevance of markers of adiposity to vascular-metabolic mortality	3
3.	Relevance of markers of adiposity to vascular-metabolic mortality, by levels of confounders	4
4.	Relevance of:	
	(a) waist circumference	5
	(b) waist-hip ratio	6
	(c) waist-height ratio	7
	(d) hip circumference	8
	to cause-specific mortality at ages 40-74 years	
5.	Independent age and sex−specific relevance of markers of adiposity to vascular-metabolic mortality	9
6.	Independent relevance of markers of adiposity to vascular-metabolic mortality, by levels of confounders	10
7.	Independent relevance of:	
	(a) waist circumference	11
	(b) waist-hip ratio	12
	(c) waist-height ratio	13
	(d) hip circumference	14
	to cause-specific mortality at ages 40-74 years	

Tables

1.	Numbers of deaths at ages 40-74 years by underlying cause (ICD-10 code)	15
2.	Characteristics of 113,163 participants aged 35-74 at recruitment, by sex and	
	(a) waist circumference	16
	(b) waist-hip ratio	17
	(c) waist-height ratio	18
	(d) hip circumference	19
	(e) BMI	20
3.	Correlation between adiposity markers in 113,163 participants aged 35-74 at recruitment	21
4.	Relevance of markers of adiposity to cause-specific vascular-metabolic mortality at ages 40-74 years, before and after mutual adjustment for other adiposity markers – sensitivity analyses	22
5.	Relevance of markers of adiposity to cause-specific non-vascular-metabolic mortality at ages 40-74 years, before and after mutual adjustment for other adiposity markers	24

Webfigure 1: Extent of regression to the mean for particular measured and derived markers of adiposity

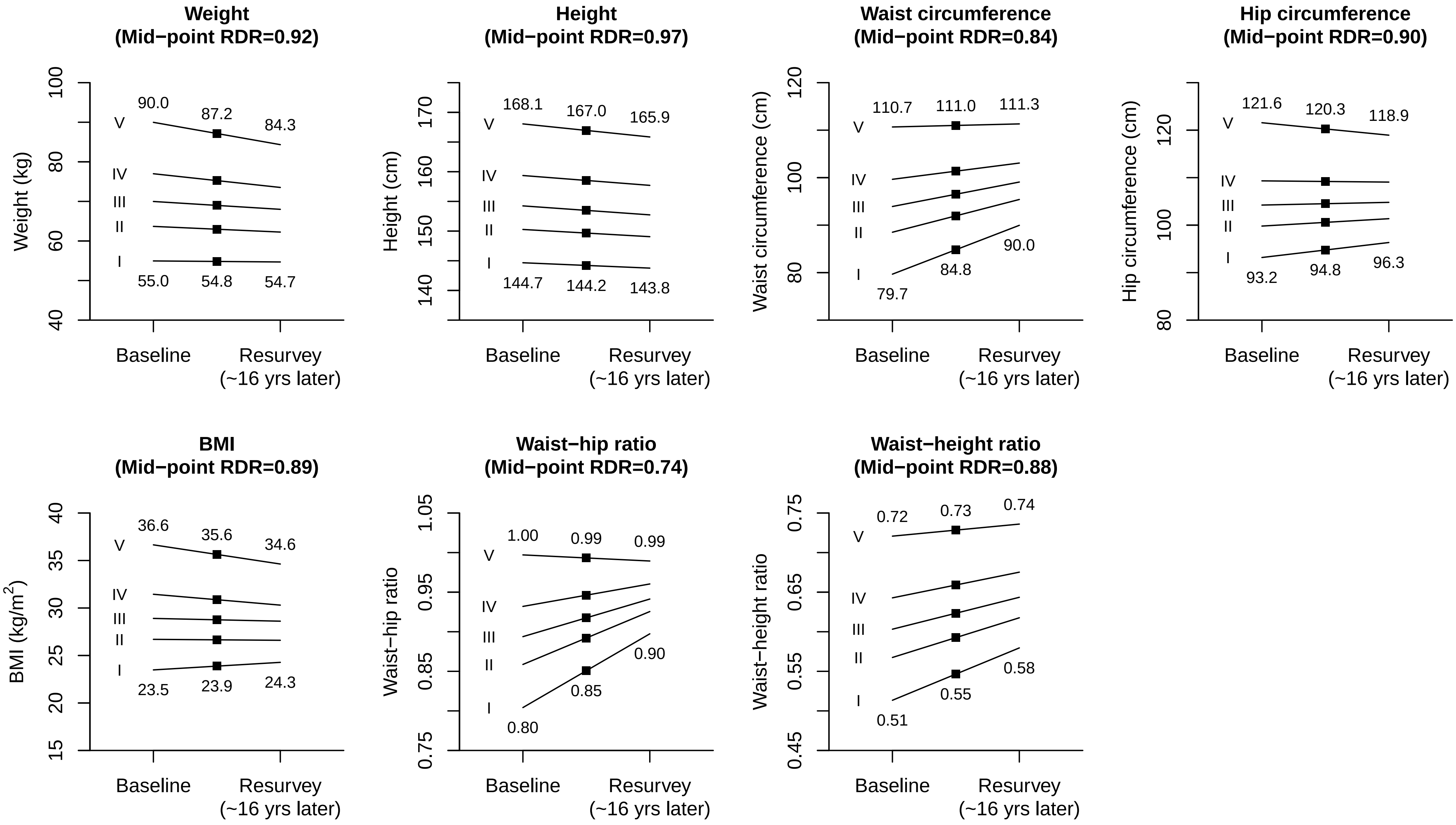

Analysis restricted to 6250 participants aged 35−74 and without diabetes or other prior chronic disease (chronic kidney disease, ischaemic heart disease, stroke, cirrhosis, cancer, or emphysema) at recruitment, and with complete, plausible, anthropometry data at both recruitment and resurvey. The regression dilution ratio (RDR) is calculated as the ratio of the range of mid−point to baseline means; for example for BMI this equates to (35.6−23.9)/(36.6−23.5)=0.89.

Webfigure 2: Age and sex−specific relevance of markers of adiposity to vascular−metabolic mortality

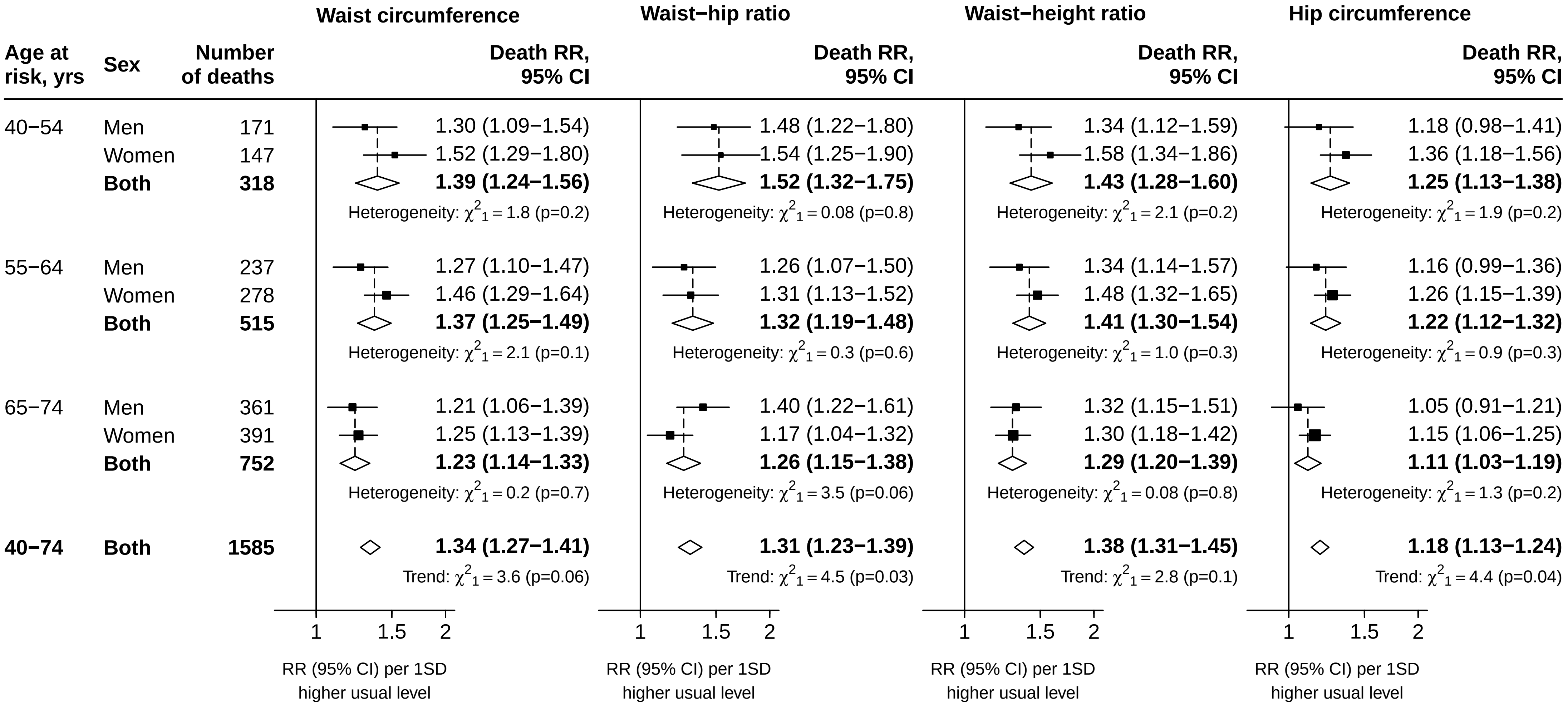

Webfigure 3: Relevance of markers of adiposity to vascular−metabolic mortality, by levels of confounders

Variable	Number of deaths	Waist circumference: Death RR, 95% CI	Waist−hip ratio: Death RR, 95% CI	Waist−height ratio: Death RR, 95% CI	Hip circumference: Death RR, 95% CI
**Age at risk (years)**					
40−54	318	1.39 (1.24−1.56)	1.52 (1.32−1.75)	1.43 (1.28−1.60)	1.25 (1.13−1.38)
55−64	515	1.37 (1.25−1.49)	1.32 (1.19−1.48)	1.41 (1.30−1.54)	1.22 (1.12−1.32)
65−74	752	1.23 (1.14−1.33)	1.26 (1.15−1.38)	1.29 (1.20−1.39)	1.11 (1.03−1.19)
**Sex**					
Male	769	1.29 (1.19−1.40)	1.36 (1.24−1.49)	1.36 (1.24−1.48)	1.11 (1.01−1.22)
Female	816	1.39 (1.29−1.49)	1.25 (1.15−1.37)	1.42 (1.33−1.52)	1.23 (1.16−1.30)
**District**					
Coyoácan	637	1.33 (1.22−1.45)	1.41 (1.27−1.55)	1.40 (1.29−1.52)	1.14 (1.05−1.23)
Iztapalapa	948	1.33 (1.24−1.42)	1.25 (1.16−1.36)	1.36 (1.28−1.45)	1.20 (1.13−1.27)
**Smoking**					
Never	652	1.42 (1.31−1.54)	1.24 (1.13−1.37)	1.43 (1.33−1.54)	1.28 (1.19−1.37)
Former	308	1.22 (1.07−1.38)	1.17 (1.01−1.35)	1.24 (1.09−1.41)	1.09 (0.98−1.22)
Current	625	1.28 (1.18−1.39)	1.47 (1.33−1.62)	1.31 (1.21−1.43)	1.09 (1.00−1.18)
**Education**					
University	190	1.44 (1.23−1.68)	1.50 (1.24−1.82)	1.51 (1.30−1.76)	1.21 (1.05−1.39)
High school	360	1.28 (1.15−1.43)	1.43 (1.25−1.63)	1.36 (1.22−1.51)	1.14 (1.04−1.26)
Elementary	869	1.35 (1.25−1.45)	1.32 (1.21−1.44)	1.38 (1.29−1.48)	1.20 (1.12−1.27)
Other	166	1.13 (0.96−1.34)	1.01 (0.84−1.22)	1.12 (0.96−1.31)	1.11 (0.96−1.27)
**Physical activity**					
None	1278	1.33 (1.25−1.41)	1.29 (1.21−1.39)	1.38 (1.30−1.46)	1.20 (1.14−1.26)
Up to twice weekly	106	1.15 (0.93−1.43)	1.46 (1.13−1.89)	1.21 (0.98−1.50)	1.09 (0.89−1.34)
At least 3 times weekly	201	1.33 (1.14−1.56)	1.47 (1.23−1.75)	1.39 (1.20−1.63)	1.07 (0.92−1.24)
**Alcohol consumption**					
None	274	1.36 (1.21−1.53)	1.26 (1.09−1.46)	1.41 (1.26−1.58)	1.20 (1.09−1.32)
Former	108	1.32 (1.08−1.62)	1.28 (1.01−1.63)	1.39 (1.13−1.70)	1.07 (0.87−1.32)
Current	1203	1.32 (1.25−1.41)	1.31 (1.22−1.41)	1.35 (1.27−1.43)	1.18 (1.12−1.25)
**Overall**	**1585**	**1.34 (1.27−1.41)**	**1.31 (1.23−1.39)**	**1.38 (1.31−1.45)**	**1.18 (1.13−1.24)**

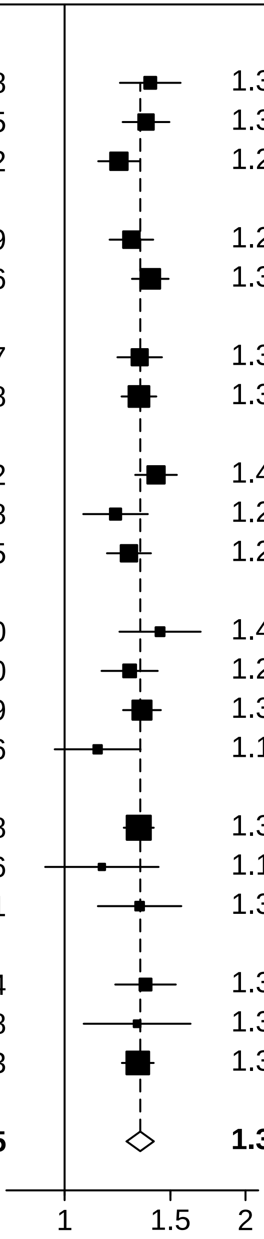

RR (95% CI) per 1SD higher usual level

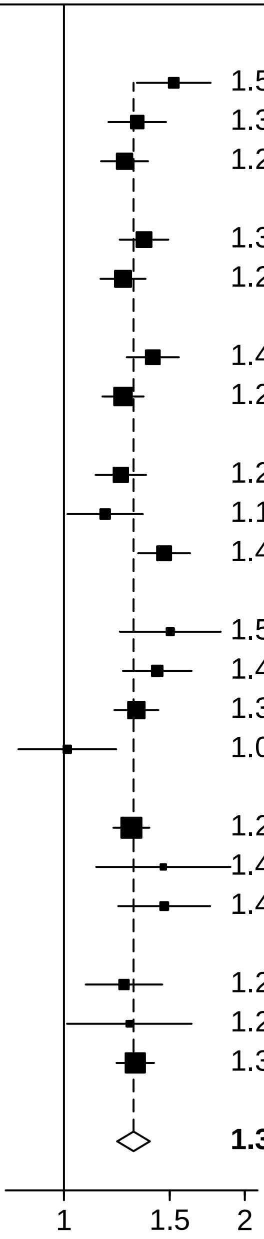

RR (95% CI) per 1SD higher usual level

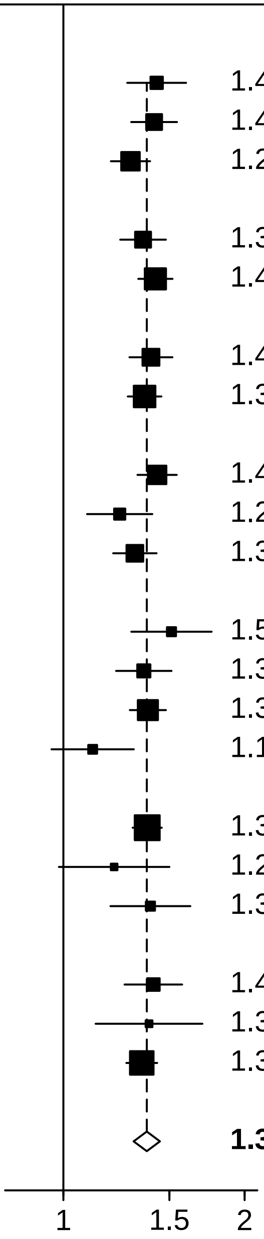

RR (95% CI) per 1SD higher usual level

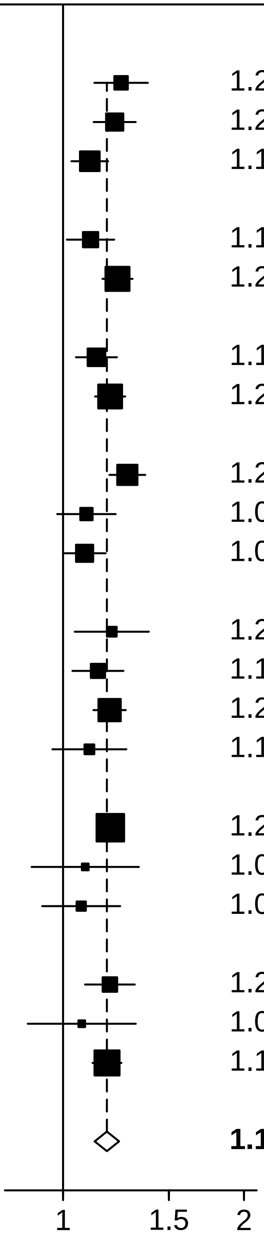

RR (95% CI) per 1SD higher usual level

Webfigure 4a: Relevance of waist circumference to cause−specific mortality at ages 40−74 years

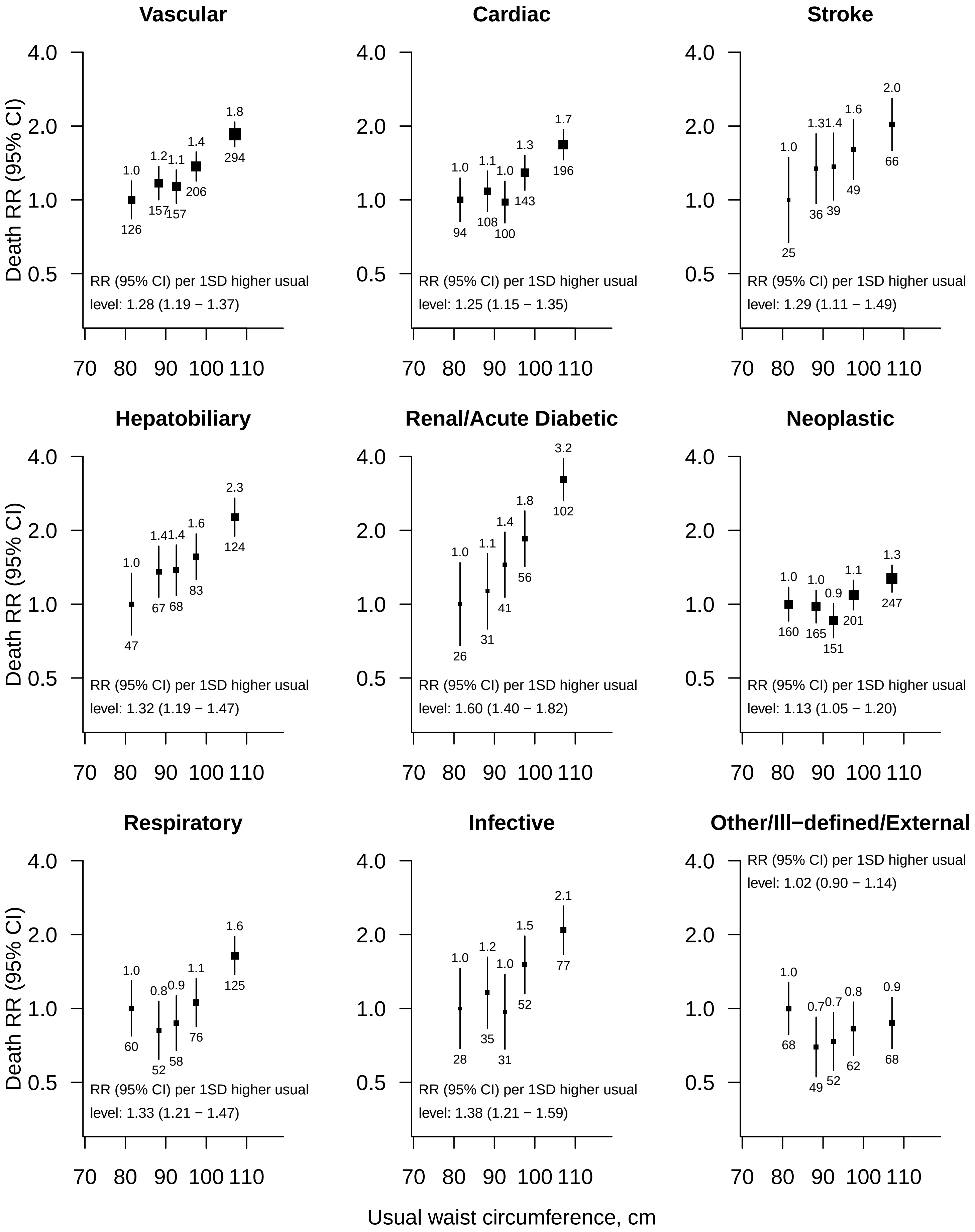

Analyses and exclusions as for Figure 1, except that the categories are now quintiles. Infective endpoint excludes respiratory infections (which are included in respiratory).

Webfigure 4b: Relevance of hip circumference to cause−specific mortality at ages 40−74 years

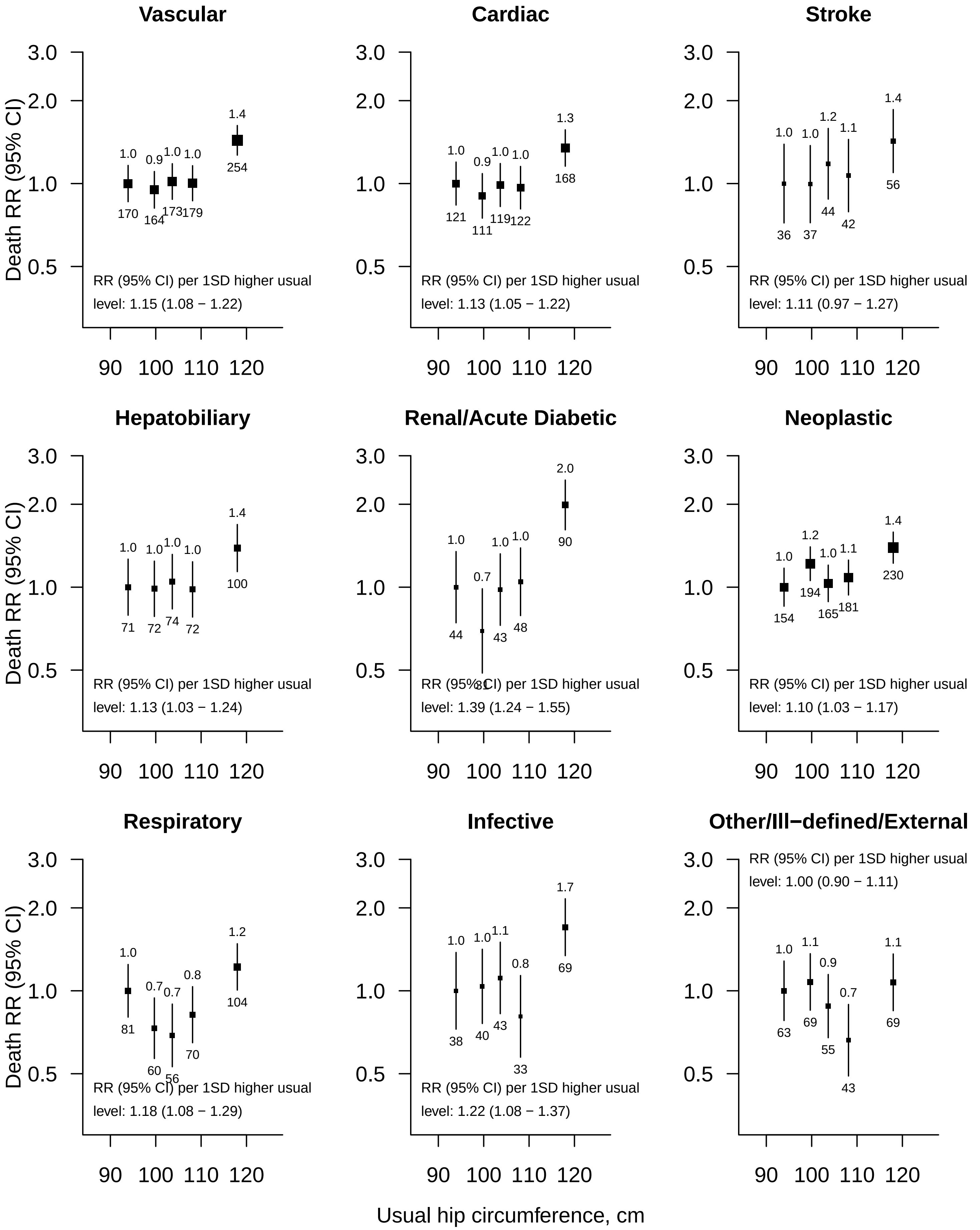

Analyses and exclusions as for Figure 1, except that the categories are now quintiles. Infective endpoint excludes respiratory infections (which are included in respiratory).

Webfigure 4c: Relevance of waist−hip ratio to cause−specific mortality at ages 40−74 years

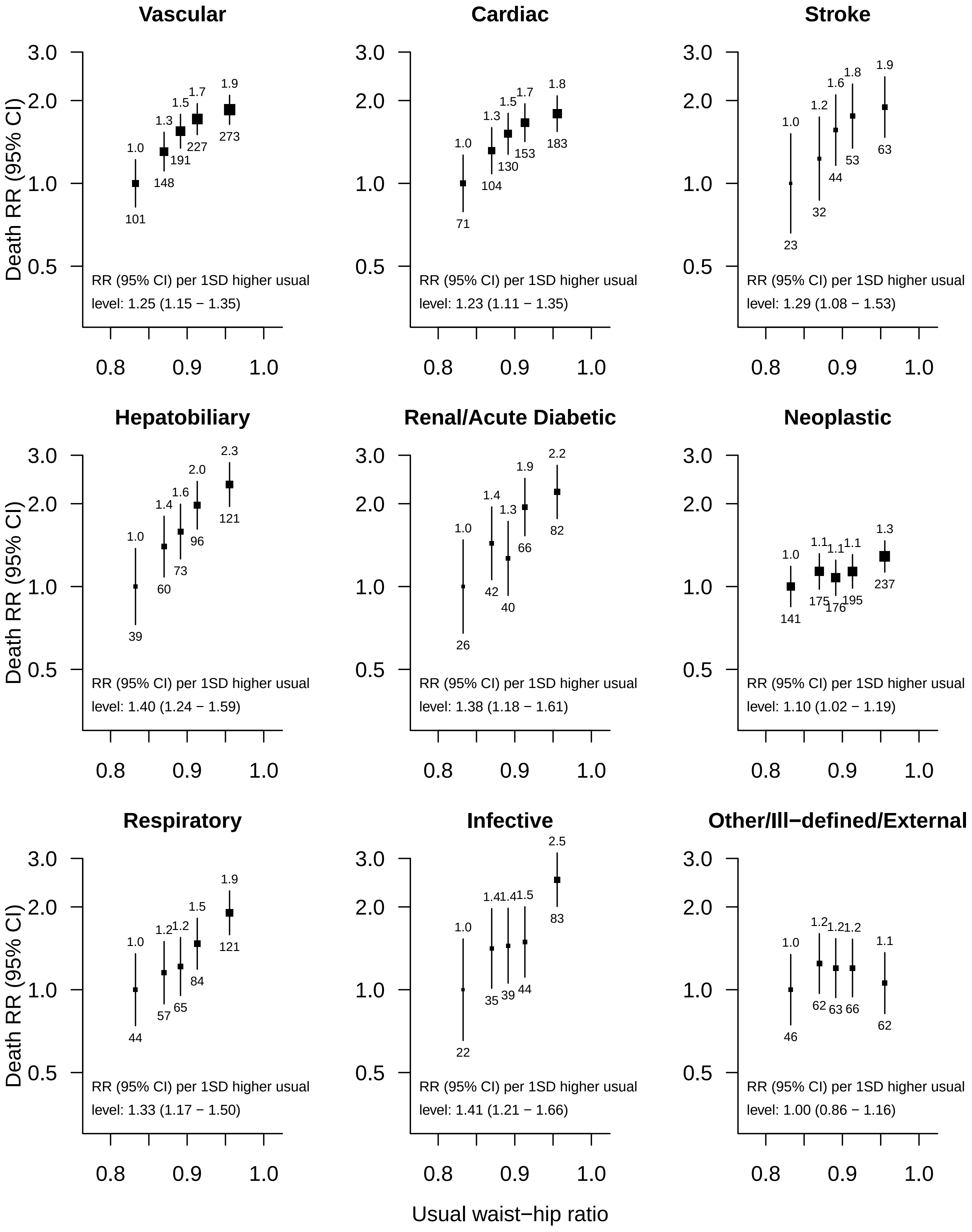

Analyses and exclusions as for Figure 1, except that the categories are now quintiles. Infective endpoint excludes respiratory infections (which are included in respiratory).

Webfigure 4d: Relevance of waist−height ratio to cause−specific mortality at ages 40−74 years

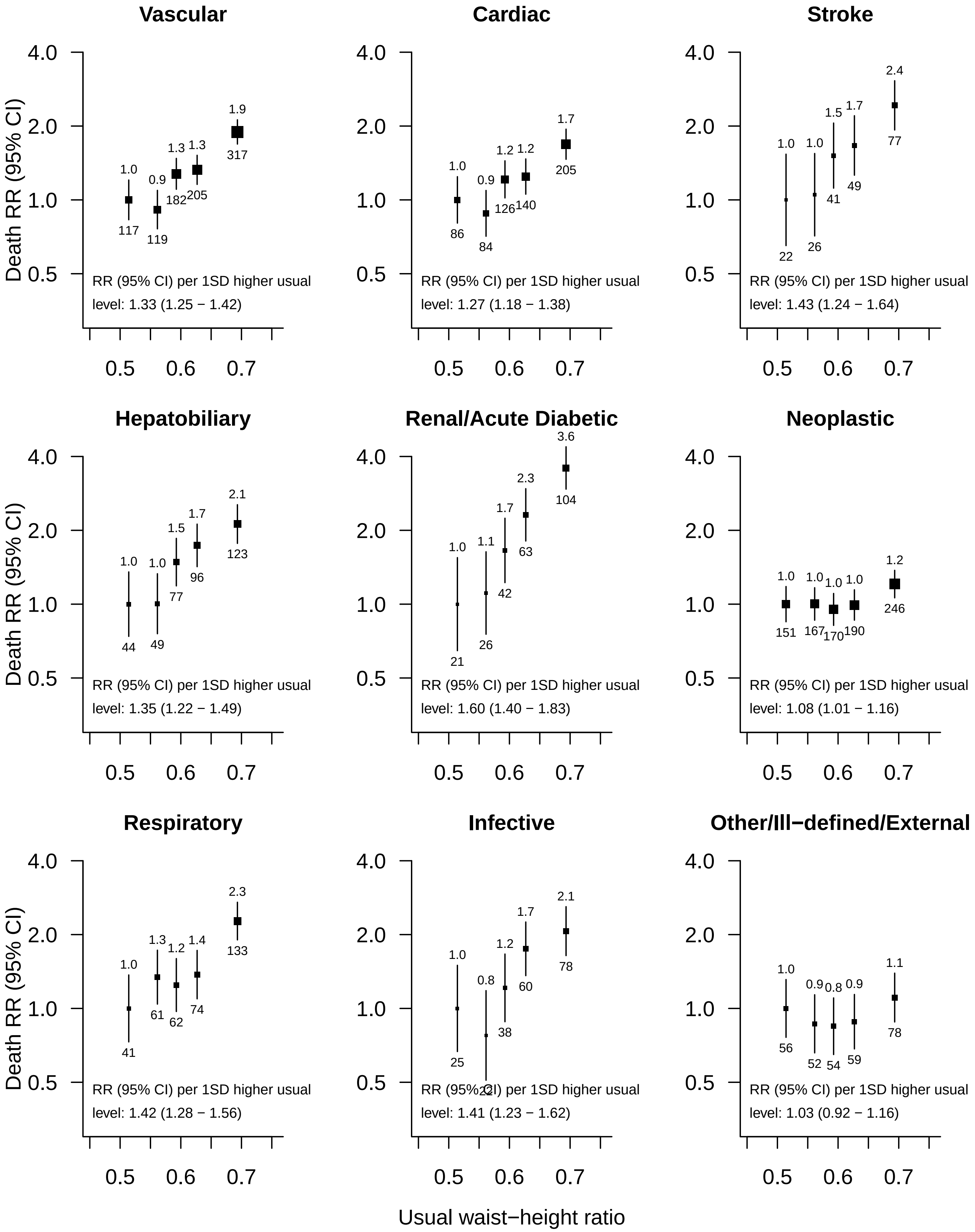

Analyses and exclusions as for Figure 1, except that the categories are now quintiles. Infective endpoint excludes respiratory infections (which are included in respiratory).

Webfigure 5: Independent age and sex−specific relevance of markers of adiposity to vascular−metabolic mortality

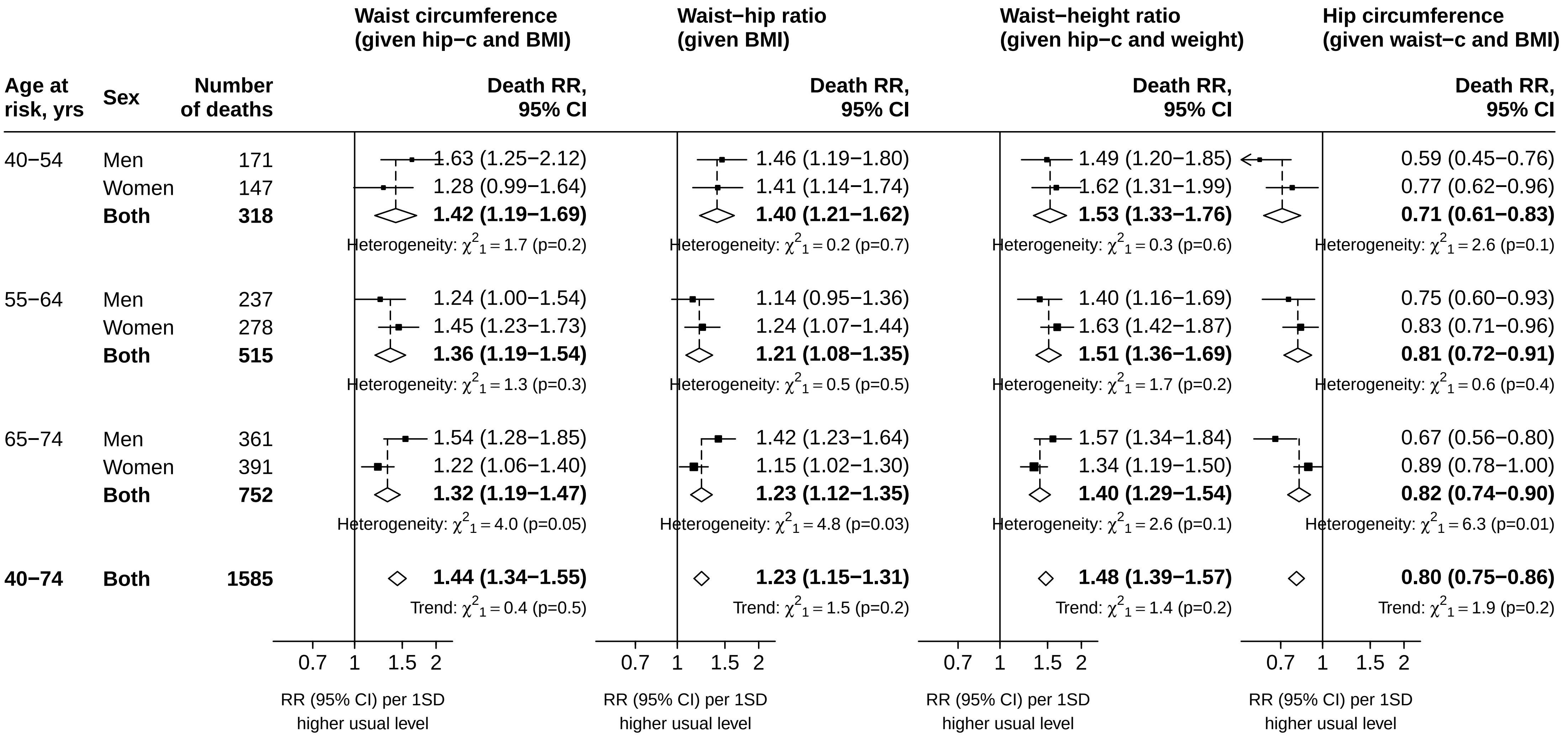

Webfigure 6: Independent relevance of markers of abdominal adiposity to vascular−metabolic mortality, by levels of confounders

Variable	Number of deaths	Waist circumference (given hip−c and BMI) Death RR, 95% CI	Waist−hip ratio (given BMI) Death RR, 95% CI	Waist−height ratio (given hip−c and weight) Death RR, 95% CI	Hip circumference (given waist−c and BMI) Death RR, 95% CI
**Age at risk (years)**					
40−54	318	1.43 (1.20−1.71)	1.40 (1.21−1.62)	1.55 (1.35−1.79)	0.74 (0.63−0.86)
55−64	515	1.34 (1.18−1.53)	1.21 (1.08−1.35)	1.50 (1.35−1.67)	0.81 (0.72−0.91)
65−74	752	1.31 (1.18−1.46)	1.22 (1.11−1.34)	1.42 (1.29−1.55)	0.84 (0.76−0.92)
**Sex**					
Male	769	1.60 (1.42−1.81)	1.31 (1.19−1.45)	1.53 (1.38−1.69)	0.66 (0.58−0.74)
Female	816	1.38 (1.25−1.52)	1.21 (1.11−1.32)	1.50 (1.38−1.62)	0.87 (0.79−0.94)
**District**					
Coyoácan	637	1.51 (1.34−1.71)	1.32 (1.19−1.47)	1.60 (1.45−1.77)	0.71 (0.64−0.80)
Iztapalapa	948	1.36 (1.23−1.49)	1.18 (1.09−1.28)	1.43 (1.32−1.54)	0.82 (0.75−0.89)
**Smoking**					
Never	652	1.34 (1.20−1.50)	1.17 (1.06−1.29)	1.42 (1.29−1.55)	0.89 (0.80−0.98)
Former	308	1.44 (1.21−1.71)	1.13 (0.98−1.32)	1.38 (1.19−1.60)	0.81 (0.69−0.95)
Current	625	1.53 (1.36−1.73)	1.42 (1.28−1.58)	1.51 (1.36−1.67)	0.66 (0.59−0.73)
**Education**					
University	190	1.62 (1.29−2.03)	1.37 (1.12−1.67)	1.78 (1.47−2.14)	0.65 (0.53−0.80)
High school	360	1.53 (1.30−1.79)	1.38 (1.21−1.58)	1.54 (1.35−1.76)	0.73 (0.63−0.85)
Elementary	869	1.35 (1.22−1.49)	1.24 (1.13−1.35)	1.47 (1.35−1.59)	0.84 (0.77−0.92)
Other	166	1.03 (0.82−1.30)	0.99 (0.82−1.19)	1.06 (0.88−1.28)	0.94 (0.77−1.16)
**Physical activity**					
None	1278	1.36 (1.25−1.48)	1.22 (1.14−1.31)	1.48 (1.38−1.58)	0.84 (0.78−0.91)
Up to twice weekly	106	1.45 (1.06−1.99)	1.50 (1.15−1.96)	1.33 (1.02−1.72)	0.76 (0.57−1.03)
At least 3 times weekly	201	1.55 (1.25−1.93)	1.35 (1.13−1.62)	1.63 (1.36−1.95)	0.57 (0.47−0.69)
**Alcohol consumption**					
None	274	1.64 (1.38−1.94)	1.22 (1.05−1.41)	1.62 (1.41−1.86)	0.79 (0.68−0.92)
Former	108	1.44 (1.06−1.95)	1.18 (0.92−1.51)	1.75 (1.36−2.23)	0.64 (0.48−0.84)
Current	1203	1.39 (1.28−1.52)	1.23 (1.15−1.33)	1.41 (1.31−1.52)	0.82 (0.76−0.88)
**Overall**	**1585**	**1.44 (1.34−1.55)**	**1.23 (1.15−1.31)**	**1.48 (1.39−1.57)**	**0.80 (0.75−0.86)**

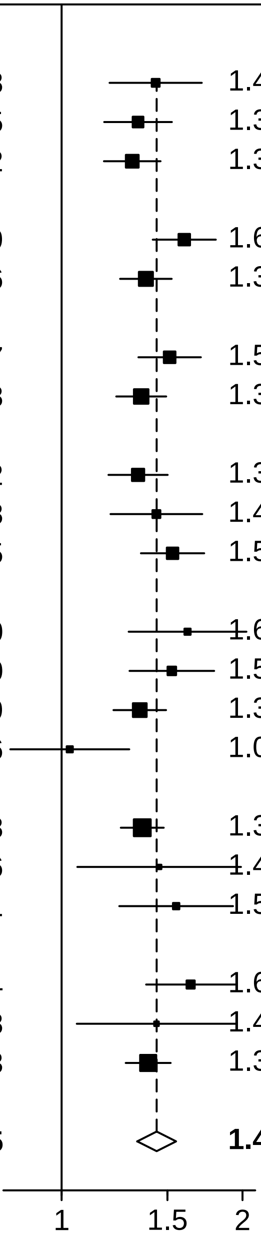

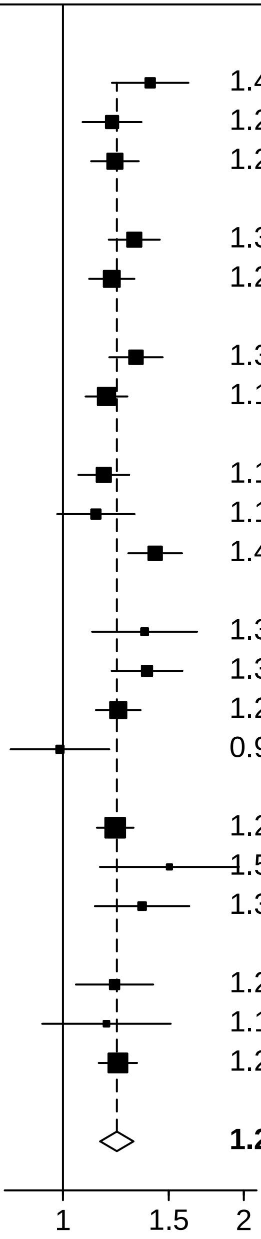

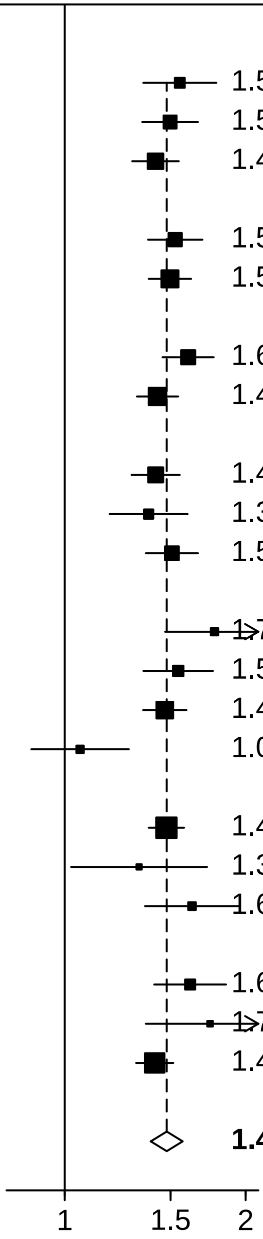

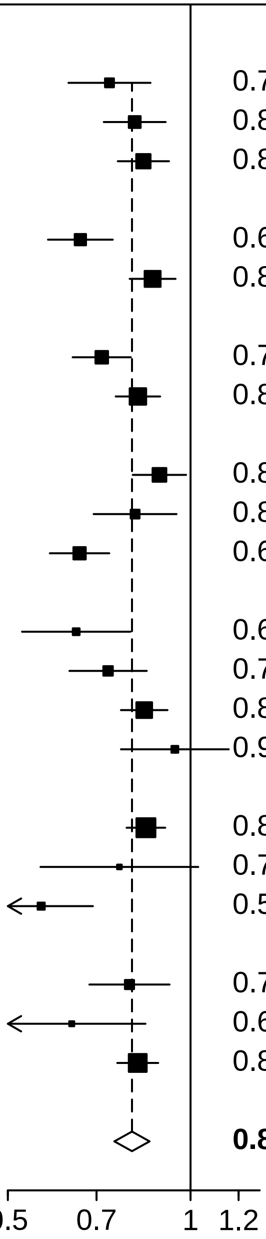

Webfigure 7a: Independent relevance of waist circumference to cause−specific mortality at ages 40−74 years

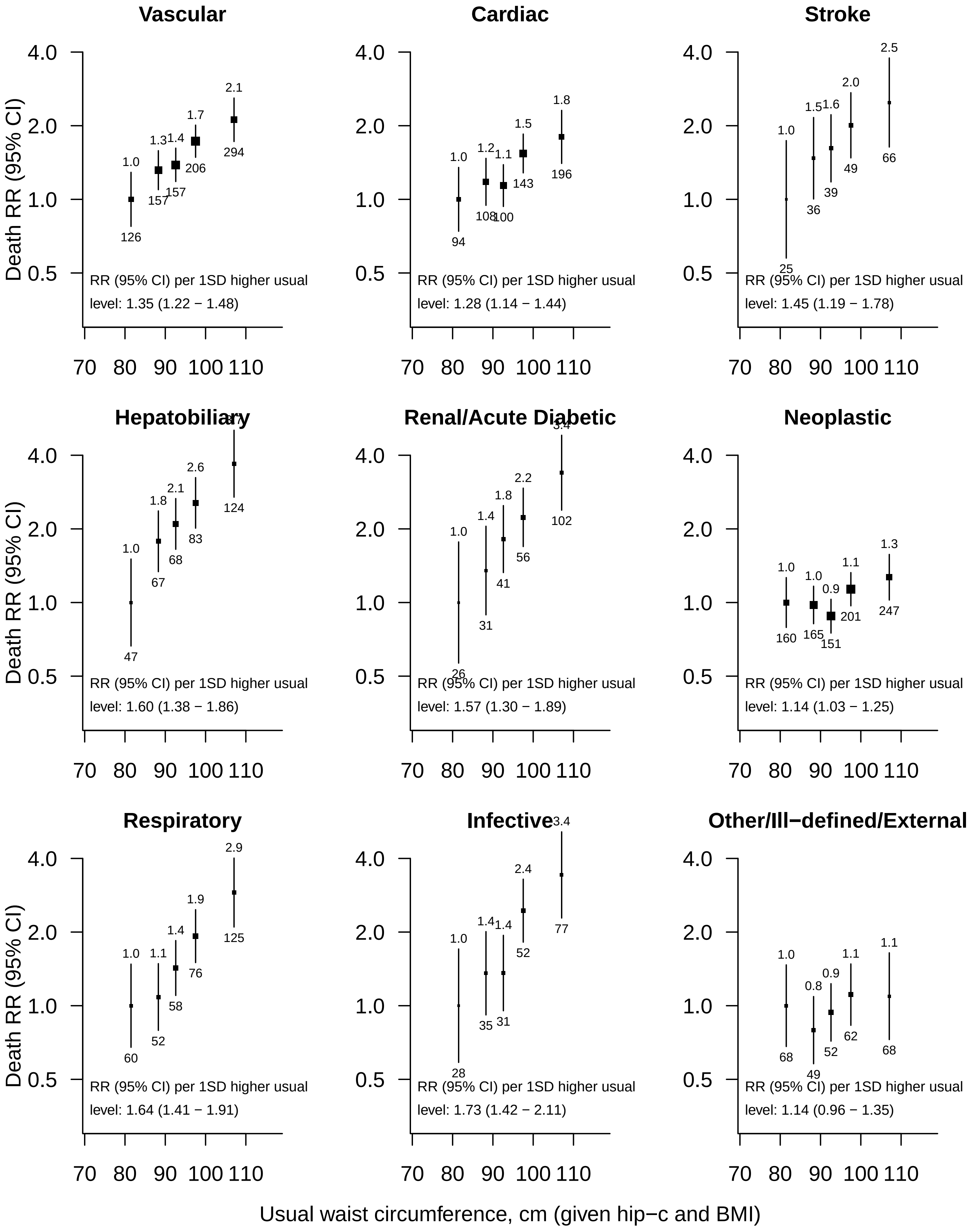

Analyses and exclusions as for Figure 2, except that the categories are now quintiles. Infective endpoint excludes respiratory infections (which are included in respiratory). Analyses further adjusted to show independent relevance,

Webfigure 7b: Independent relevance of hip circumference to cause−specific mortality at ages 40−74 years

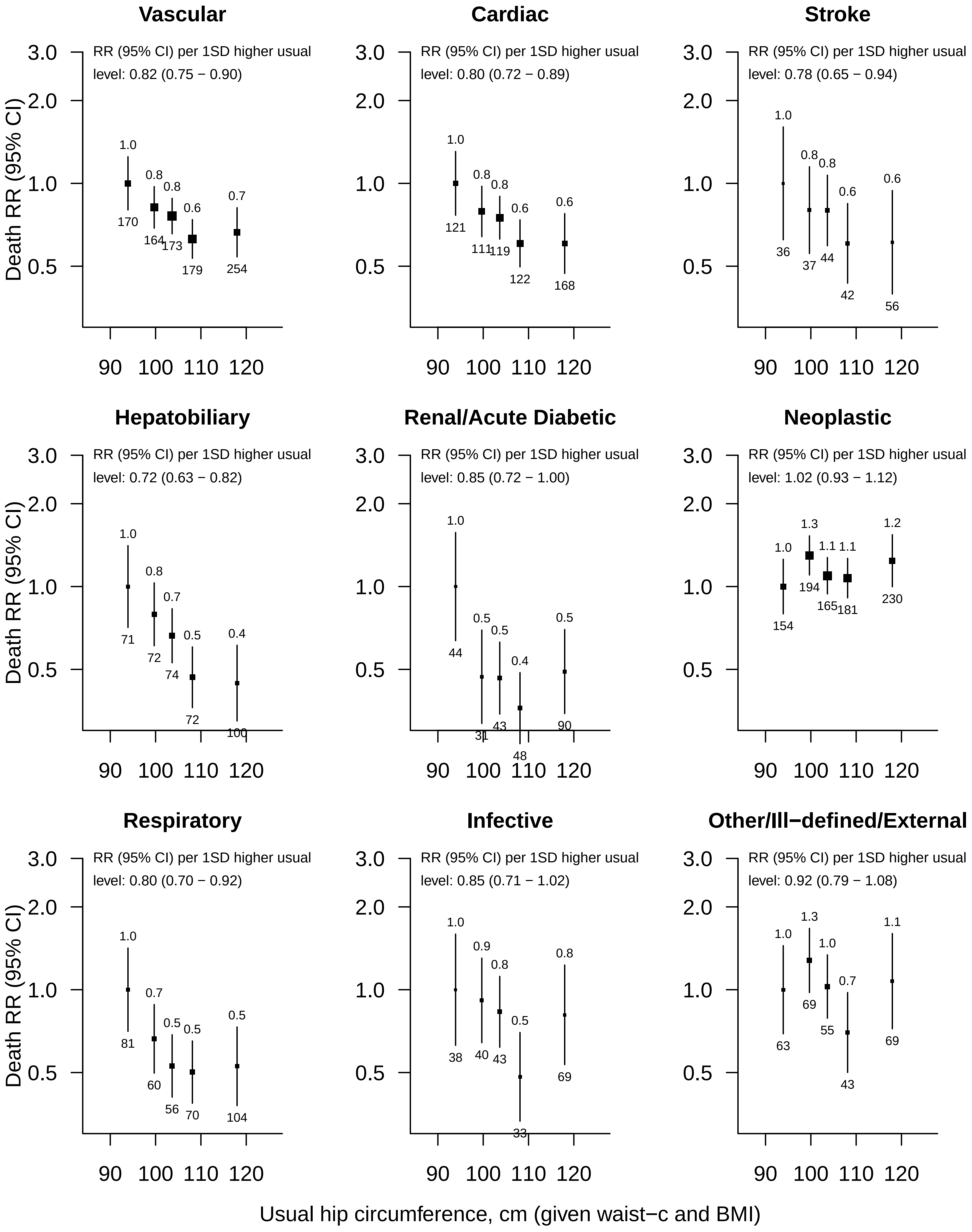

Analyses and exclusions as for Figure 2, except that the categories are now quintiles. Infective endpoint excludes respiratory infections (which are included in respiratory). Analyses further adjusted to show independent relevance.

Webfigure 7c: Independent relevance of waist−hip ratio to cause−specific mortality at ages 40−74 years

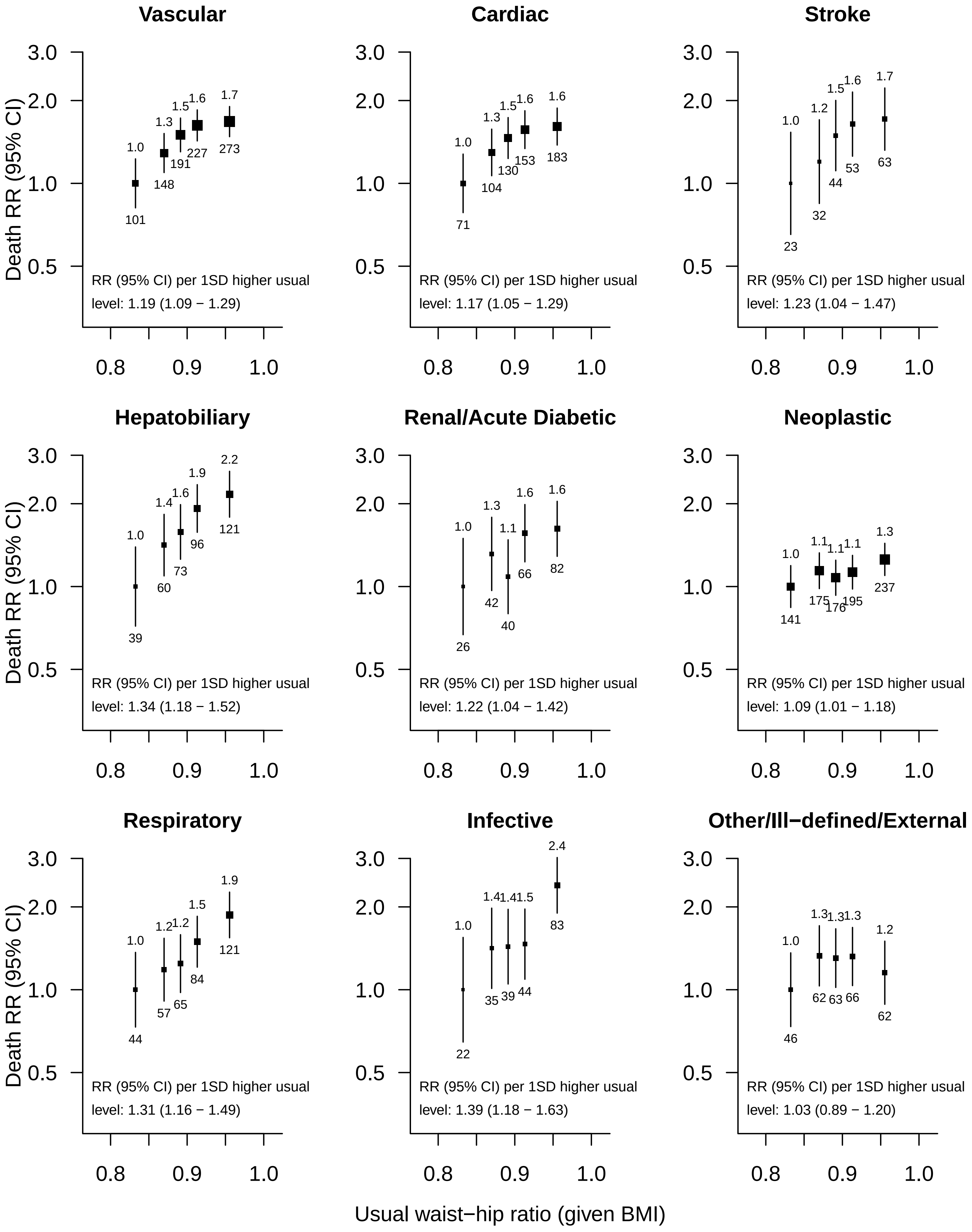

Analyses and exclusions as for Figure 2, except that the categories are now quintiles. Infective endpoint excludes respiratory infections (which are included in respiratory). Analyses further adjusted to show independent relevance.

Webfigure 7d: Independent relevance of waist−height ratio to cause−specific mortality at ages 40−74 years

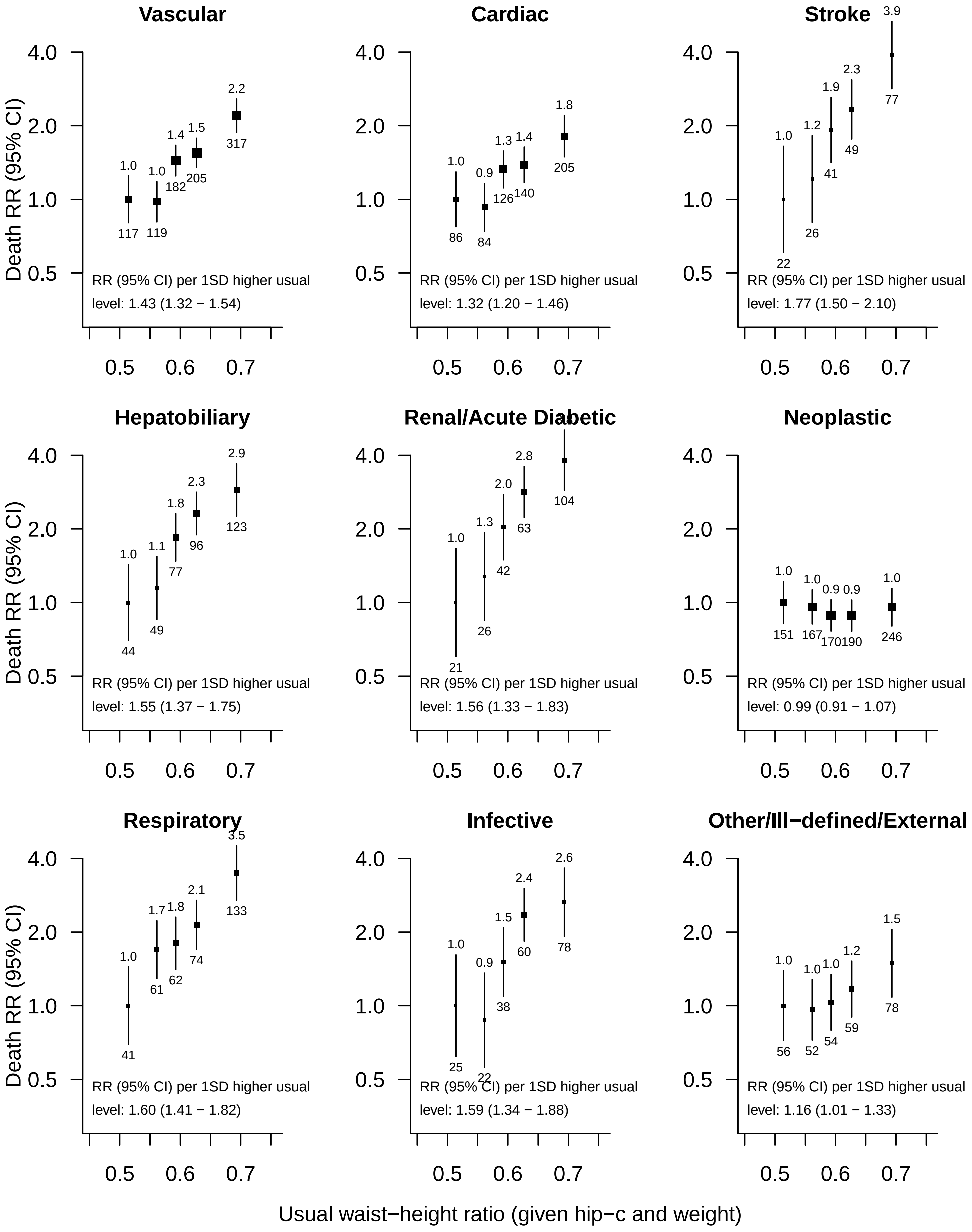

Analyses and exclusions as for Figure 2, except that the categories are now quintiles. Infective endpoint excludes respiratory infections (which are included in respiratory). Analyses further adjusted to show independent relevance.

Webtable 1: Numbers of deaths at ages 40-74 years by underlying cause (ICD-10 code)

Underlying cause of death	ICD-10 codes (and number of deaths)
Cardiac (n=641)	I051 (2), I059 (6), I070 (1), I071 (2), I080 (1), I081 (1), I091 (1), I099 (11), I110 (28), I119 (6), I200 (1), I209 (1), I210 (1), I211 (4), I219 (454), I221 (1), I249 (1), I251 (8), I258 (2), I259 (22), I270 (5), I272 (1), I301 (1), I330 (3), I340 (3), I350 (7), I351 (1), I358 (1), I38X (1), I420 (3), I429 (1), I442 (1), I471 (1), I472 (1), I489 (2), I48X (3), I490 (3), I500 (19), I501 (3), I509 (14), I518 (1), I519 (3), Q210 (1), Q231 (1), Q238 (1), Q248 (1), R570 (5)
Stroke (n=215)	I600 (1), I609 (27), I610 (1), I615 (1), I619 (68), I620 (4), I629 (3), I634 (6), I639 (16), I64X (37), I671 (6), I673 (1), I678 (19), I679 (19), I694 (2), I698 (4)
Other vascular (n=84)	E115 (2), E145 (1), I260 (1), I269 (24), I710 (3), I712 (1), I713 (3), I718 (1), I719 (1), I729 (1), I739 (1), I741 (1), I771 (1), I776 (1), I802 (3), I822 (1), I828 (1), I829 (1), I872 (4), I879 (1), I99X (1), K550 (24), K559 (5), K761 (1)
Renal (n=208)	E102 (1), E112 (34), E142 (15), I120 (30), I129 (2), I131 (1), I132 (3), N002 (1), N039 (4), N059 (2), N12X (2), N151 (3), N179 (23), N180 (5), N189 (30), N19X (4), N200 (4), N201 (1), N289 (1), N390 (42)
Acute diabetic crisis (n=48)	E110 (10), E111 (13), E140 (9), E141 (15), E162 (1)
Hepatobiliary (n=389)	B169 (1), B171 (8), B181 (1), B182 (6), B189 (1), B190 (2), I850 (7), I859 (3), K701 (12), K702 (1), K703 (56), K704 (7), K709 (9), K716 (1), K720 (4), K721 (26), K729 (79), K745 (1), K746 (95), K750 (3), K754 (2), K766 (2), K767 (4), K769 (5), K800 (3), K801 (4), K803 (3), K805 (1), K810 (5), K811 (2), K819 (2), K821 (1), K829 (3), K830 (9), K831 (1), K852 (3), K858 (1), K859 (11), K85X (2), K861 (1), Q447 (1)
Neoplastic (n=924)	C029 (5), C069 (1), C07X (1), C089 (1), C109 (2), C119 (2), C139 (1), C140 (2), C159 (6), C169 (81), C170 (4), C179 (1), C187 (2), C189 (55), C19X (2), C20X (5), C220 (24), C221 (7), C229 (40), C23X (7), C240 (3), C241 (4), C249 (6), C250 (4), C259 (40), C260 (2), C319 (1), C329 (6), C33X (1), C349 (72), C37X (1), C383 (1), C384 (1), C402 (1), C412 (1), C414 (1), C419 (5), C439 (9), C445 (1), C447 (1), C449 (5), C450 (1), C451 (1), C459 (1), C469 (1), C479 (1), C480 (5), C482 (4), C492 (1), C499 (5), C509 (96), C519 (1), C539 (41), C541 (8), C549 (1), C55X (6), C56X (51), C61X (32), C629 (1), C64X (30), C679 (6), C680 (3), C709 (1), C710 (7), C711 (1), C719 (20), C73X (13), C749 (1), C751 (1), C760 (3), C764 (1), C779 (1), C780 (3), C787 (5), C788 (1), C794 (1), C795 (1), C796 (1), C798 (1), C800 (14), C809 (11), C80X (1), C817 (1), C819 (5), C833 (6), C839 (1), C844 (1), C845 (1), C851 (1), C859 (23), C900 (24), C910 (11), C911 (1), C919 (1), C920 (14), C921 (5), C927 (1), C929 (1), C959 (1), C97X (1), D371 (1), D372 (1), D374 (2), D376 (4), D377 (1), D381 (2), D391 (2), D410 (1), D430 (5), D449 (1), D484 (1), D487 (4), D489 (1)
Respiratory* (n=371)	A162 (1), B206 (2), J09 (2), J100 (2), J157 (1), J159 (11), J180 (20), J181 (8), J189 (154), J209 (2), J22X (3), J348 (1), J42X (4), J439 (10), J440 (26), J448 (2), J449 (53), J459 (4), J64X (3), J679 (2), J680 (1), J80X (1), J81X (2), J841 (38), J849 (6), J852 (1), J980 (1), J984 (6), J985 (1), J988 (2), Q311 (1)
Infective (n=223)	A047 (2), A090 (4), A099 (13), A09X (2), A181 (1), A182 (1), A183 (1), A188 (1), A199 (2), A415 (1), A419 (45), A86X (1), B201 (1), B207 (2), B208 (3), B210 (1), B212 (1), B238 (2), B24X (1), B690 (2), G049 (2), K052 (1), K223 (1), K228 (1), K251 (3), K254 (3), K255 (3), K256 (1), K259 (3), K264 (3), K265 (1), K269 (1), K274 (1), K290 (1), K291 (1), K295 (2), K318 (2), K353 (2), K358 (1), K572 (1), K578 (3), K579 (5), K610 (1), K611 (1), K650 (4), K659 (18), K920 (5), K921 (1), K922 (36), L021 (1), L031 (1), L039 (2), L089 (4), L899 (1), L905 (1), L984 (2), M009 (1), M726 (1), M798 (10), N410 (1), N498 (1), N719 (1), N739 (1)

*Includes deaths from respiratory infection

Webtable 2a: Characteristics of 113 163 participants aged 35-74 at recruitment, by sex and waist circumference

	Men							
	Waist circumference group* (participants)							
	I (3 695)	II (3 912)	III (7 450)	IV (7 036)	V (7 364)	VI (3 595)	VII (3 594)	Total (36 646)
**Age, socio-economic status and lifestyle behaviours**								
Age, years	47 (10)	48 (10)	49 (10)	50 (11)	51 (11)	52 (11)	52 (11)	50 (11)
Resident of Coyoacán	1797 (49%)	1958 (50%)	3429 (46%)	3181 (45%)	3210 (44%)	1495 (42%)	1427 (40%)	16 497 (45%)
University/college educated	986 (27%)	1192 (30%)	2137 (29%)	1992 (28%)	1859 (25%)	893 (25%)	919 (26%)	9978 (27%)
Current smoker	2194 (59%)	2160 (55%)	3978 (53%)	3570 (51%)	3707 (50%)	1762 (49%)	1723 (48%)	19 094 (52%)
Current drinker	3111 (84%)	3338 (85%)	6326 (85%)	5986 (85%)	6232 (85%)	3052 (85%)	3021 (84%)	31 066 (85%)
Any regular leisure-time physical activity	1361 (37%)	1431 (37%)	2532 (34%)	2258 (32%)	2148 (29%)	966 (27%)	812 (23%)	11 508 (31%)
**Physical measurements**								
Height, cm	163 (7)	164 (7)	164 (7)	165 (7)	166 (7)	166 (7)	167 (7)	165 (7)
Weight, kg	61 (7)	66 (6)	71 (6)	75 (7)	81 (7)	87 (8)	97 (12)	76 (12)
BMI, kg/m²	23.0 (2.4)	24.8 (2.1)	26.2 (2.1)	27.7 (2.2)	29.4 (2.4)	31.3 (2.5)	34.8 (4.0)	28.0 (4.1)
Waist circumference, cm	80 (4)	86 (1)	91 (1)	95 (1)	100 (2)	106 (1)	116 (8)	96 (10)
Hip circumference, cm	92 (5)	95 (4)	98 (4)	101 (4)	104 (5)	107 (5)	114 (8)	101 (8)
Waist-hip ratio	0.87 (0.05)	0.91 (0.04)	0.93 (0.04)	0.95 (0.04)	0.97 (0.04)	0.99 (0.04)	1.02 (0.06)	0.95 (0.06)
Waist-height ratio	0.49 (0.03)	0.53 (0.02)	0.55 (0.02)	0.58 (0.02)	0.61 (0.03)	0.64 (0.03)	0.69 (0.05)	0.58 (0.06)
SBP, mmHg	122 (13)	124 (14)	125 (14)	127 (14)	129 (14)	131 (15)	134 (16)	127 (15)
DBP, mmHg	81 (9)	82 (9)	83 (9)	84 (9)	85 (9)	86 (10)	88 (10)	84 (10)
**Glycated haemoglobin**								
Mean (SD), %	5.3 (0.3)	5.3 (0.3)	5.4 (0.3)	5.4 (0.4)	5.5 (0.4)	5.6 (0.4)	5.6 (0.4)	5.4 (0.4)
**Long term medication use**								
Any anti-hypertensive	92 (2%)	150 (4%)	362 (5%)	479 (7%)	674 (9%)	433 (12%)	562 (16%)	2752 (8%)
Any anti-thrombotic	48 (1%)	68 (2%)	91 (1%)	133 (2%)	197 (3%)	106 (3%)	87 (2%)	730 (2%)
Any lipid lowering	10 (<0.5%)	18 (<0.5%)	21 (<0.5%)	32 (<0.5%)	39 (1%)	16 (<0.5%)	13 (<0.5%)	149 (<0.5%)

	Women							
	Waist circumference group* (participants)							
	I (7 701)	II (7 620)	III (15 314)	IV (15 387)	V (15 215)	VI (7 697)	VII (7 583)	Total (76 517)
**Age, socio-economic status and lifestyle behaviours**								
Age, years	45 (9)	46 (9)	47 (10)	49 (10)	50 (11)	52 (11)	52 (11)	49 (10)
Resident of Coyoacán	4015 (52%)	3699 (49%)	6397 (42%)	5772 (38%)	5427 (36%)	2586 (34%)	2425 (32%)	30 321 (40%)
University/college educated	2056 (27%)	1537 (20%)	2377 (16%)	1940 (13%)	1472 (10%)	629 (8%)	594 (8%)	10 605 (14%)
Current smoker	2443 (32%)	2189 (29%)	4010 (26%)	3766 (24%)	3458 (23%)	1663 (22%)	1718 (23%)	19 247 (25%)
Current drinker	5459 (71%)	5466 (72%)	10 938 (71%)	10 908 (71%)	10 644 (70%)	5304 (69%)	5279 (70%)	53 998 (71%)
Any regular leisure-time physical activity	2062 (27%)	1886 (25%)	3423 (22%)	2909 (19%)	2406 (16%)	1075 (14%)	949 (13%)	14 710 (19%)
**Physical measurements**								
Height, cm	152 (6)	152 (6)	152 (6)	152 (6)	152 (6)	152 (6)	152 (6)	152 (6)
Weight, kg	54 (6)	59 (6)	63 (7)	67 (7)	72 (8)	78 (9)	88 (12)	68 (12)
BMI, kg/m²	23.3 (2.4)	25.4 (2.3)	27.1 (2.5)	29.0 (2.7)	31.2 (3.1)	33.8 (3.4)	37.9 (4.9)	29.5 (5.0)
Waist circumference, cm	74 (4)	81 (1)	86 (2)	91 (2)	98 (2)	104 (2)	115 (7)	92 (12)
Hip circumference, cm	93 (5)	97 (5)	101 (6)	105 (6)	110 (7)	115 (7)	125 (11)	106 (11)
Waist-hip ratio	0.79 (0.05)	0.83 (0.04)	0.85 (0.05)	0.87 (0.05)	0.89 (0.05)	0.91 (0.06)	0.93 (0.07)	0.87 (0.06)
Waist-height ratio	0.49 (0.03)	0.53 (0.02)	0.56 (0.03)	0.60 (0.03)	0.64 (0.03)	0.69 (0.03)	0.75 (0.05)	0.61 (0.08)
SBP, mmHg	117 (14)	120 (14)	122 (15)	124 (15)	127 (16)	129 (16)	133 (16)	124 (16)
DBP, mmHg	77 (9)	79 (10)	80 (10)	82 (10)	83 (10)	85 (10)	87 (10)	82 (10)
**Glycated haemoglobin**								
Mean (SD), %	5.2 (0.3)	5.3 (0.3)	5.4 (0.3)	5.5 (0.4)	5.5 (0.4)	5.6 (0.4)	5.7 (0.4)	5.5 (0.4)
**Long term medication use**								
Any anti-hypertensive	382 (5%)	469 (6%)	1423 (9%)	1811 (12%)	2282 (15%)	1478 (19%)	1855 (24%)	9700 (13%)
Any anti-thrombotic	146 (2%)	139 (2%)	352 (2%)	358 (2%)	429 (3%)	270 (4%)	245 (3%)	1939 (3%)
Any lipid lowering	23 (<0.5%)	41 (1%)	59 (<0.5%)	64 (<0.5%)	59 (<0.5%)	33 (<0.5%)	35 (<0.5%)	314 (<0.5%)

Conventions and exclusions as per Table 1. * Groupings of waist circumference are the top and bottom two-tenths and middle three-fifths of the sex-specific baseline distribution.

Webtable 2b: Characteristics of 113 163 participants aged 35-74 at recruitment, by sex and waist-hip ratio

	Men							
	Waist-hip ratio group* (participants)							
	I (3 664)	II (3 665)	III (7 338)	IV (7 321)	V (7 372)	VI (3 622)	VII (3 664)	Total (36 646)
**Age, socio-economic status and lifestyle behaviours**								
Age, years	45 (9)	46 (9)	48 (10)	50 (10)	52 (11)	53 (11)	55 (11)	50 (11)
Resident of Coyoacán	1820 (50%)	1782 (49%)	3567 (49%)	3323 (45%)	3179 (43%)	1485 (41%)	1341 (37%)	16 497 (45%)
University/college educated	1243 (34%)	1242 (34%)	2263 (31%)	2100 (29%)	1763 (24%)	755 (21%)	612 (17%)	9978 (27%)
Current smoker	2050 (56%)	2019 (55%)	3874 (53%)	3804 (52%)	3746 (51%)	1820 (50%)	1781 (49%)	19 094 (52%)
Current drinker	3075 (84%)	3102 (85%)	6245 (85%)	6228 (85%)	6235 (85%)	3070 (85%)	3111 (85%)	31 066 (85%)
Any regular leisure-time physical activity	1559 (43%)	1427 (39%)	2585 (35%)	2245 (31%)	2015 (27%)	905 (25%)	772 (21%)	11 508 (31%)
**Physical measurements**								
Height, cm	165 (7)	166 (7)	165 (7)	165 (7)	165 (7)	165 (7)	164 (7)	165 (7)
Weight, kg	68 (11)	71 (10)	74 (11)	76 (11)	79 (12)	82 (13)	85 (15)	76 (12)
BMI, kg/m²	24.9 (3.5)	25.9 (3.1)	27.0 (3.3)	27.9 (3.4)	28.9 (3.6)	30.2 (4.1)	31.3 (4.5)	28.0 (4.1)
Waist circumference, cm	84 (8)	89 (6)	92 (6)	96 (7)	99 (7)	104 (8)	109 (10)	96 (10)
Hip circumference, cm	99 (10)	99 (6)	101 (7)	101 (7)	102 (7)	103 (8)	103 (9)	101 (8)
Waist-hip ratio	0.85 (0.04)	0.89 (0.01)	0.92 (0.01)	0.95 (0.01)	0.97 (0.01)	1.00 (0.01)	1.05 (0.05)	0.95 (0.06)
Waist-height ratio	0.51 (0.05)	0.54 (0.04)	0.56 (0.04)	0.58 (0.04)	0.60 (0.04)	0.63 (0.05)	0.66 (0.06)	0.58 (0.06)
SBP, mmHg	122 (13)	124 (13)	125 (14)	127 (14)	129 (15)	131 (16)	132 (16)	127 (15)
DBP, mmHg	81 (9)	82 (9)	83 (9)	84 (9)	85 (10)	86 (10)	86 (10)	84 (10)
**Glycated haemoglobin**								
Mean (SD), %	5.3 (0.3)	5.4 (0.3)	5.4 (0.3)	5.4 (0.4)	5.5 (0.4)	5.5 (0.4)	5.6 (0.4)	5.4 (0.4)
**Long term medication use**								
Any anti-hypertensive	127 (3%)	149 (4%)	385 (5%)	529 (7%)	684 (9%)	405 (11%)	473 (13%)	2752 (8%)
Any anti-thrombotic	46 (1%)	54 (1%)	117 (2%)	149 (2%)	162 (2%)	91 (3%)	111 (3%)	730 (2%)
Any lipid lowering	11 (<0.5%)	11 (<0.5%)	33 (<0.5%)	30 (<0.5%)	33 (<0.5%)	17 (<0.5%)	14 (<0.5%)	149 (<0.5%)

	Women							
	Waist-hip ratio group* (participants)							
	I (7 651)	II (7 642)	III (15 385)	IV (15 371)	V (15 226)	VI (7 588)	VII (7 654)	Total (76 517)
**Age, socio-economic status and lifestyle behaviours**								
Age, years	46 (9)	46 (9)	47 (10)	48 (10)	50 (10)	52 (11)	54 (12)	49 (10)
Resident of Coyoacán	4131 (54%)	3619 (47%)	6304 (41%)	5791 (38%)	5298 (35%)	2647 (35%)	2531 (33%)	30 321 (40%)
University/college educated	2110 (28%)	1566 (20%)	2408 (16%)	1903 (12%)	1451 (10%)	630 (8%)	537 (7%)	10 605 (14%)
Current smoker	2410 (31%)	2192 (29%)	3988 (26%)	3864 (25%)	3586 (24%)	1692 (22%)	1515 (20%)	19 247 (25%)
Current drinker	5588 (73%)	5590 (73%)	10 983 (71%)	10 871 (71%)	10 602 (70%)	5237 (69%)	5127 (67%)	53 998 (71%)
Any regular leisure-time physical activity	2251 (29%)	1790 (23%)	3164 (21%)	2818 (18%)	2452 (16%)	1131 (15%)	1104 (14%)	14 710 (19%)
**Physical measurements**								
Height, cm	154 (6)	153 (6)	152 (6)	152 (6)	151 (6)	151 (6)	151 (7)	152 (6)
Weight, kg	63 (12)	65 (12)	67 (12)	69 (12)	70 (12)	71 (12)	71 (13)	68 (12)
BMI, kg/m²	26.7 (5.0)	27.8 (4.8)	28.9 (4.8)	29.8 (4.9)	30.5 (4.8)	31.0 (4.9)	31.0 (5.0)	29.5 (5.0)
Waist circumference, cm	80 (9)	85 (9)	89 (9)	93 (9)	96 (9)	100 (10)	104 (11)	92 (12)
Hip circumference, cm	105 (13)	105 (11)	106 (11)	107 (11)	107 (10)	107 (11)	105 (11)	106 (11)
Waist-hip ratio	0.76 (0.03)	0.81 (0.01)	0.84 (0.01)	0.87 (0.01)	0.90 (0.01)	0.93 (0.01)	0.99 (0.05)	0.87 (0.06)
Waist-height ratio	0.52 (0.06)	0.55 (0.06)	0.58 (0.06)	0.61 (0.06)	0.64 (0.06)	0.66 (0.07)	0.69 (0.07)	0.61 (0.08)
SBP, mmHg	120 (14)	121 (14)	123 (15)	124 (15)	126 (16)	128 (16)	129 (17)	124 (16)
DBP, mmHg	79 (10)	80 (10)	81 (10)	82 (10)	83 (10)	84 (10)	84 (10)	82 (10)
**Glycated haemoglobin**								
Mean (SD), %	5.3 (0.3)	5.3 (0.3)	5.4 (0.4)	5.5 (0.4)	5.5 (0.4)	5.6 (0.4)	5.6 (0.4)	5.5 (0.4)
**Long term medication use**								
Any anti-hypertensive	607 (8%)	697 (9%)	1584 (10%)	1896 (12%)	2170 (14%)	1282 (17%)	1464 (19%)	9700 (13%)
Any anti-thrombotic	168 (2%)	174 (2%)	380 (2%)	406 (3%)	386 (3%)	213 (3%)	212 (3%)	1939 (3%)
Any lipid lowering	17 (<0.5%)	31 (<0.5%)	69 (<0.5%)	73 (<0.5%)	64 (<0.5%)	30 (<0.5%)	30 (<0.5%)	314 (<0.5%)

Conventions and exclusions as per Table 1. * Groupings of waist-hip ratio are the top and bottom two-tenths and middle three-fifths of the sex-specific baseline distribution.

Webtable 2c: Characteristics of 113 163 participants aged 35-74 at recruitment, by sex and waist-height ratio

	Men							
	Waist-height ratio group* (participants)							
	I (3 664)	II (3 665)	III (7 331)	IV (7 333)	V (7 331)	VI (3 657)	VII (3 665)	Total (36 646)
**Age, socio-economic status and lifestyle behaviours**								
Age, years	46 (9)	47 (10)	48 (10)	50 (11)	52 (11)	53 (11)	54 (11)	50 (11)
Resident of Coyoacán	1907 (52%)	1830 (50%)	3490 (48%)	3315 (45%)	3101 (42%)	1466 (40%)	1388 (38%)	16 497 (45%)
University/college educated	1289 (35%)	1279 (35%)	2331 (32%)	1963 (27%)	1730 (24%)	722 (20%)	664 (18%)	9978 (27%)
Current smoker	2223 (61%)	2099 (57%)	3926 (54%)	3809 (52%)	3590 (49%)	1752 (48%)	1695 (46%)	19 094 (52%)
Current drinker	3072 (84%)	3120 (85%)	6233 (85%)	6202 (85%)	6227 (85%)	3143 (86%)	3069 (84%)	31 066 (85%)
Any regular leisure-time physical activity	1465 (40%)	1396 (38%)	2606 (36%)	2340 (32%)	2025 (28%)	927 (25%)	749 (20%)	11 508 (31%)
**Physical measurements**								
Height, cm	168 (7)	167 (7)	166 (7)	165 (7)	164 (7)	163 (7)	162 (7)	165 (7)
Weight, kg	65 (9)	69 (8)	72 (9)	76 (9)	79 (10)	84 (11)	92 (15)	76 (12)
BMI, kg/m²	22.9 (2.3)	24.6 (2.0)	26.1 (2.0)	27.7 (2.1)	29.4 (2.3)	31.3 (2.4)	34.8 (4.0)	28.0 (4.1)
Waist circumference, cm	81 (5)	87 (4)	91 (4)	95 (4)	100 (4)	105 (4)	114 (9)	96 (10)
Hip circumference, cm	93 (5)	96 (5)	99 (5)	101 (5)	103 (6)	106 (6)	112 (9)	101 (8)
Waist-hip ratio	0.87 (0.05)	0.91 (0.04)	0.93 (0.04)	0.95 (0.04)	0.97 (0.04)	0.99 (0.04)	1.02 (0.06)	0.95 (0.06)
Waist-height ratio	0.48 (0.02)	0.52 (0.01)	0.55 (0.01)	0.58 (0.01)	0.61 (0.01)	0.64 (0.01)	0.70 (0.05)	0.58 (0.06)
SBP, mmHg	121 (13)	123 (13)	125 (14)	127 (14)	129 (15)	131 (15)	134 (16)	127 (15)
DBP, mmHg	81 (9)	82 (9)	83 (9)	84 (9)	85 (9)	86 (10)	88 (10)	84 (10)
**Glycated haemoglobin**								
Mean (SD), %	5.3 (0.3)	5.3 (0.3)	5.4 (0.3)	5.4 (0.3)	5.5 (0.4)	5.6 (0.4)	5.6 (0.4)	5.4 (0.4)
**Long term medication use**								
Any anti-hypertensive	78 (2%)	121 (3%)	360 (5%)	475 (6%)	690 (9%)	475 (13%)	553 (15%)	2752 (8%)
Any anti-thrombotic	46 (1%)	49 (1%)	116 (2%)	127 (2%)	187 (3%)	110 (3%)	95 (3%)	730 (2%)
Any lipid lowering	10 (<0.5%)	8 (<0.5%)	30 (<0.5%)	42 (1%)	28 (<0.5%)	20 (1%)	11 (<0.5%)	149 (<0.5%)

	Women							
	Waist-height ratio group* (participants)							
	I (7 651)	II (7 655)	III (15 300)	IV (15 305)	V (15 301)	VI (7 655)	VII (7 650)	Total (76 517)
**Age, socio-economic status and lifestyle behaviours**								
Age, years	44 (8)	45 (9)	47 (9)	49 (10)	51 (11)	53 (11)	54 (11)	49 (10)
Resident of Coyoacán	4131 (54%)	3592 (47%)	6436 (42%)	5758 (38%)	5385 (35%)	2576 (34%)	2443 (32%)	30 321 (40%)
University/college educated	2417 (32%)	1667 (22%)	2475 (16%)	1855 (12%)	1243 (8%)	514 (7%)	434 (6%)	10 605 (14%)
Current smoker	2672 (35%)	2319 (30%)	4166 (27%)	3680 (24%)	3451 (23%)	1467 (19%)	1492 (20%)	19 247 (25%)
Current drinker	5530 (72%)	5532 (72%)	10 966 (72%)	10 848 (71%)	10 675 (70%)	5197 (68%)	5250 (69%)	53 998 (71%)
Any regular leisure-time physical activity	2289 (30%)	1944 (25%)	3371 (22%)	2868 (19%)	2298 (15%)	1021 (13%)	919 (12%)	14 710 (19%)
**Physical measurements**								
Height, cm	156 (6)	154 (6)	153 (6)	152 (6)	151 (6)	150 (6)	149 (6)	152 (6)
Weight, kg	56 (7)	60 (8)	64 (8)	67 (9)	71 (10)	76 (11)	84 (14)	68 (12)
BMI, kg/m²	23.3 (2.3)	25.3 (2.2)	27.1 (2.4)	29.1 (2.7)	31.2 (3.1)	33.8 (3.4)	37.8 (4.9)	29.5 (5.0)
Waist circumference, cm	75 (4)	81 (3)	86 (4)	91 (4)	97 (4)	104 (4)	114 (8)	92 (12)
Hip circumference, cm	95 (6)	98 (6)	101 (6)	105 (7)	109 (8)	114 (9)	123 (11)	106 (11)
Waist-hip ratio	0.79 (0.05)	0.83 (0.04)	0.85 (0.04)	0.87 (0.05)	0.89 (0.05)	0.91 (0.06)	0.93 (0.07)	0.87 (0.06)
Waist-height ratio	0.48 (0.02)	0.53 (0.01)	0.56 (0.01)	0.60 (0.01)	0.65 (0.01)	0.69 (0.01)	0.76 (0.05)	0.61 (0.08)
SBP, mmHg	117 (13)	119 (14)	121 (14)	124 (15)	127 (16)	130 (16)	133 (17)	124 (16)
DBP, mmHg	77 (9)	79 (9)	80 (9)	82 (10)	83 (10)	85 (10)	87 (10)	82 (10)
**Glycated haemoglobin**								
Mean (SD), %	5.2 (0.3)	5.3 (0.3)	5.4 (0.3)	5.5 (0.4)	5.5 (0.4)	5.6 (0.4)	5.7 (0.4)	5.5 (0.4)
**Long term medication use**								
Any anti-hypertensive	358 (5%)	473 (6%)	1287 (8%)	1765 (12%)	2378 (16%)	1500 (20%)	1939 (25%)	9700 (13%)
Any anti-thrombotic	149 (2%)	152 (2%)	330 (2%)	359 (2%)	451 (3%)	241 (3%)	257 (3%)	1939 (3%)
Any lipid lowering	22 (<0.5%)	33 (<0.5%)	77 (1%)	54 (<0.5%)	64 (<0.5%)	35 (<0.5%)	29 (<0.5%)	314 (<0.5%)

Conventions and exclusions as per Table 1. * Groupings of waist-height ratio are the top and bottom two-tenths and middle three-fifths of the sex-specific baseline distribution.

Webtable 2d: Characteristics of 113 163 participants aged 35-74 at recruitment, by sex and hip circumference

	Men							
	Hip circumference group* (participants)							
	I (3 711)	II (3 646)	III (7 794)	IV (6 970)	V (7 230)	VI (3 668)	VII (3 627)	Total (36 646)
**Age, socio-economic status and lifestyle behaviours**								
Age, years	50 (11)	50 (11)	50 (11)	50 (11)	50 (10)	50 (10)	50 (11)	50 (11)
Resident of Coyoacán	1718 (46%)	1690 (46%)	3553 (46%)	3079 (44%)	3332 (46%)	1589 (43%)	1536 (42%)	16 497 (45%)
University/college educated	774 (21%)	837 (23%)	2093 (27%)	1930 (28%)	2116 (29%)	1096 (30%)	1132 (31%)	9978 (27%)
Current smoker	2193 (59%)	1976 (54%)	4134 (53%)	3578 (51%)	3673 (51%)	1779 (49%)	1761 (49%)	19 094 (52%)
Current drinker	3167 (85%)	3127 (86%)	6588 (85%)	5918 (85%)	6092 (84%)	3095 (84%)	3079 (85%)	31 066 (85%)
Any regular leisure-time physical activity	1131 (30%)	1134 (31%)	2541 (33%)	2262 (32%)	2363 (33%)	1134 (31%)	943 (26%)	11 508 (31%)
**Physical measurements**								
Height, cm	161 (7)	163 (6)	164 (6)	165 (6)	167 (7)	168 (7)	168 (7)	165 (7)
Weight, kg	60 (7)	66 (6)	70 (6)	76 (6)	81 (7)	87 (8)	97 (12)	76 (12)
BMI, kg/m²	23.4 (2.6)	25.0 (2.3)	26.2 (2.3)	27.8 (2.5)	29.3 (2.6)	31.0 (2.7)	34.4 (4.2)	28.0 (4.1)
Waist circumference, cm	84 (6)	88 (5)	91 (5)	95 (6)	100 (6)	104 (6)	113 (10)	96 (10)
Hip circumference, cm	90 (3)	94 (1)	97 (1)	101 (1)	104 (1)	108 (1)	117 (8)	101 (8)
Waist-hip ratio	0.93 (0.07)	0.93 (0.06)	0.94 (0.05)	0.95 (0.05)	0.95 (0.05)	0.96 (0.06)	0.97 (0.07)	0.95 (0.06)
Waist-height ratio	0.52 (0.05)	0.54 (0.04)	0.56 (0.04)	0.58 (0.04)	0.60 (0.05)	0.62 (0.05)	0.67 (0.07)	0.58 (0.06)
SBP, mmHg	123 (15)	125 (14)	126 (15)	127 (14)	128 (14)	130 (15)	132 (16)	127 (15)
DBP, mmHg	81 (9)	83 (9)	83 (9)	84 (9)	85 (9)	86 (10)	87 (10)	84 (10)
**Glycated haemoglobin**								
Mean (SD), %	5.4 (0.3)	5.4 (0.3)	5.4 (0.3)	5.5 (0.4)	5.5 (0.4)	5.5 (0.4)	5.6 (0.4)	5.4 (0.4)
**Long term medication use**								
Any anti-hypertensive	122 (3%)	189 (5%)	454 (6%)	480 (7%)	613 (8%)	363 (10%)	531 (15%)	2752 (8%)
Any anti-thrombotic	54 (1%)	64 (2%)	138 (2%)	148 (2%)	143 (2%)	90 (2%)	93 (3%)	730 (2%)
Any lipid lowering	11 (<0.5%)	16 (<0.5%)	33 (<0.5%)	21 (<0.5%)	40 (1%)	15 (<0.5%)	13 (<0.5%)	149 (<0.5%)

	Women							
	Hip circumference group* (participants)							
	I (8 125)	II (7 211)	III (15 370)	IV (15 291)	V (15 548)	VI (7 426)	VII (7 546)	Total (76 517)
**Age, socio-economic status and lifestyle behaviours**								
Age, years	47 (11)	47 (10)	48 (10)	49 (10)	50 (10)	51 (11)	51 (10)	49 (10)
Resident of Coyoacán	3480 (43%)	3235 (45%)	6507 (42%)	6021 (39%)	5878 (38%)	2644 (36%)	2556 (34%)	30 321 (40%)
University/college educated	1458 (18%)	1265 (18%)	2457 (16%)	2093 (14%)	1849 (12%)	740 (10%)	743 (10%)	10 605 (14%)
Current smoker	2189 (27%)	1973 (27%)	4038 (26%)	3854 (25%)	3722 (24%)	1705 (23%)	1766 (23%)	19 247 (25%)
Current drinker	5526 (68%)	5125 (71%)	10 903 (71%)	10 899 (71%)	10 921 (70%)	5293 (71%)	5331 (71%)	53 998 (71%)
Any regular leisure-time physical activity	1706 (21%)	1650 (23%)	3363 (22%)	3080 (20%)	2714 (17%)	1192 (16%)	1005 (13%)	14 710 (19%)
**Physical measurements**								
Height, cm	150 (6)	151 (6)	152 (6)	152 (6)	153 (6)	153 (6)	153 (6)	152 (6)
Weight, kg	53 (6)	58 (5)	62 (6)	67 (6)	73 (7)	79 (7)	90 (11)	68 (12)
BMI, kg/m²	23.6 (2.6)	25.3 (2.4)	26.9 (2.5)	28.9 (2.6)	31.2 (2.8)	33.9 (2.9)	38.5 (4.5)	29.5 (5.0)
Waist circumference, cm	78 (7)	83 (6)	87 (6)	91 (6)	97 (7)	102 (7)	112 (9)	92 (12)
Hip circumference, cm	91 (3)	96 (1)	100 (1)	105 (1)	110 (2)	117 (2)	129 (8)	106 (11)
Waist-hip ratio	0.86 (0.08)	0.86 (0.06)	0.87 (0.06)	0.87 (0.06)	0.87 (0.06)	0.88 (0.06)	0.87 (0.06)	0.87 (0.06)
Waist-height ratio	0.52 (0.05)	0.55 (0.05)	0.57 (0.05)	0.60 (0.05)	0.63 (0.05)	0.67 (0.06)	0.73 (0.07)	0.61 (0.08)
SBP, mmHg	119 (15)	121 (15)	122 (15)	124 (15)	126 (15)	128 (16)	132 (16)	124 (16)
DBP, mmHg	78 (10)	79 (10)	80 (10)	82 (10)	83 (10)	84 (10)	86 (10)	82 (10)
**Glycated haemoglobin**								
Mean (SD), %	5.3 (0.3)	5.3 (0.4)	5.4 (0.4)	5.4 (0.4)	5.5 (0.4)	5.6 (0.4)	5.6 (0.4)	5.5 (0.4)
**Long term medication use**								
Any anti-hypertensive	548 (7%)	569 (8%)	1469 (10%)	1807 (12%)	2235 (14%)	1322 (18%)	1750 (23%)	9700 (13%)
Any anti-thrombotic	155 (2%)	154 (2%)	328 (2%)	369 (2%)	418 (3%)	249 (3%)	266 (4%)	1939 (3%)
Any lipid lowering	32 (<0.5%)	30 (<0.5%)	64 (<0.5%)	58 (<0.5%)	66 (<0.5%)	38 (1%)	26 (<0.5%)	314 (<0.5%)

Conventions and exclusions as per Table 1. * Groupings of hip circumference are the top and bottom two-tenths and middle three-fifths of the sex-specific baseline distribution.

Webtable 2e: Characteristics of 113 163 participants aged 35-74 at recruitment, by sex and BMI

	Men							
	BMI group* (participants)							
	I (3 664)	II (3 662)	III (7 332)	IV (7 331)	V (7 333)	VI (3 660)	VII (3 664)	Total (36 646)
**Age, socio-economic status and lifestyle behaviours**								
Age, years	50 (11)	50 (11)	50 (11)	50 (11)	50 (11)	50 (10)	49 (10)	50 (11)
Resident of Coyoacán	1864 (51%)	1851 (51%)	3560 (49%)	3306 (45%)	3092 (42%)	1437 (39%)	1387 (38%)	16 497 (45%)
University/college educated	1030 (28%)	1115 (30%)	2202 (30%)	2035 (28%)	1915 (26%)	843 (23%)	838 (23%)	9978 (27%)
Current smoker	2205 (60%)	1995 (54%)	3853 (53%)	3724 (51%)	3644 (50%)	1843 (50%)	1830 (50%)	19 094 (52%)
Current drinker	3079 (84%)	3118 (85%)	6226 (85%)	6243 (85%)	6192 (84%)	3104 (85%)	3104 (85%)	31 066 (85%)
Any regular leisure-time physical activity	1197 (33%)	1252 (34%)	2533 (35%)	2461 (34%)	2221 (30%)	992 (27%)	852 (23%)	11 508 (31%)
**Physical measurements**								
Height, cm	166 (7)	166 (7)	165 (7)	165 (7)	165 (7)	165 (7)	164 (7)	165 (7)
Weight, kg	60 (6)	66 (6)	70 (6)	75 (6)	81 (7)	87 (7)	98 (12)	76 (12)
BMI, kg/m²	21.7 (1.2)	24.0 (0.4)	25.8 (0.6)	27.7 (0.5)	29.7 (0.7)	31.9 (0.6)	36.0 (3.0)	28.0 (4.1)
Waist circumference, cm	83 (6)	88 (6)	91 (5)	95 (5)	99 (6)	104 (6)	112 (10)	96 (10)
Hip circumference, cm	92 (5)	96 (4)	98 (5)	101 (5)	104 (5)	107 (6)	113 (9)	101 (8)
Waist-hip ratio	0.90 (0.06)	0.92 (0.05)	0.93 (0.05)	0.95 (0.05)	0.96 (0.05)	0.98 (0.06)	0.99 (0.06)	0.95 (0.06)
Waist-height ratio	0.50 (0.04)	0.53 (0.03)	0.55 (0.03)	0.58 (0.03)	0.60 (0.04)	0.63 (0.04)	0.68 (0.06)	0.58 (0.06)
SBP, mmHg	123 (14)	124 (14)	125 (14)	127 (14)	128 (14)	130 (15)	133 (16)	127 (15)
DBP, mmHg	81 (10)	82 (9)	83 (9)	84 (9)	85 (9)	86 (10)	88 (10)	84 (10)
**Glycated haemoglobin**								
Mean (SD), %	5.3 (0.3)	5.4 (0.3)	5.4 (0.3)	5.4 (0.4)	5.5 (0.4)	5.6 (0.4)	5.6 (0.4)	5.4 (0.4)
**Long term medication use**								
Any anti-hypertensive	112 (3%)	176 (5%)	411 (6%)	545 (7%)	644 (9%)	380 (10%)	484 (13%)	2752 (8%)
Any anti-thrombotic	59 (2%)	46 (1%)	133 (2%)	159 (2%)	160 (2%)	93 (3%)	80 (2%)	730 (2%)
Any lipid lowering	6 (<0.5%)	21 (1%)	17 (<0.5%)	40 (1%)	43 (1%)	14 (<0.5%)	8 (<0.5%)	149 (<0.5%)

	Women							
	BMI group* (participants)							
	I (7 667)	II (7 641)	III (15 279)	IV (15 325)	V (15 270)	VI (7 680)	VII (7 655)	Total (76 517)
**Age, socio-economic status and lifestyle behaviours**								
Age, years	47 (11)	47 (10)	48 (10)	49 (10)	50 (10)	50 (10)	50 (10)	49 (10)
Resident of Coyoacán	3730 (49%)	3592 (47%)	6538 (43%)	5903 (39%)	5469 (36%)	2643 (34%)	2446 (32%)	30 321 (40%)
University/college educated	1907 (25%)	1532 (20%)	2463 (16%)	1918 (13%)	1511 (10%)	638 (8%)	636 (8%)	10 605 (14%)
Current smoker	2291 (30%)	2141 (28%)	4113 (27%)	3707 (24%)	3535 (23%)	1718 (22%)	1742 (23%)	19 247 (25%)
Current drinker	5301 (69%)	5409 (71%)	10 918 (71%)	10 783 (70%)	10 777 (71%)	5397 (70%)	5413 (71%)	53 998 (71%)
Any regular leisure-time physical activity	1863 (24%)	1902 (25%)	3455 (23%)	2950 (19%)	2486 (16%)	1084 (14%)	970 (13%)	14 710 (19%)
**Physical measurements**								
Height, cm	154 (7)	153 (6)	153 (6)	152 (6)	151 (6)	151 (6)	151 (6)	152 (6)
Weight, kg	52 (5)	58 (5)	62 (5)	67 (5)	72 (6)	79 (6)	90 (11)	68 (12)
BMI, kg/m²	22.1 (1.2)	24.5 (0.5)	26.6 (0.7)	28.9 (0.7)	31.6 (0.9)	34.5 (0.8)	39.7 (3.5)	29.5 (5.0)
Waist circumference, cm	78 (7)	83 (6)	87 (6)	91 (6)	97 (7)	102 (7)	111 (10)	92 (12)
Hip circumference, cm	93 (5)	97 (5)	101 (5)	105 (6)	110 (6)	115 (7)	125 (10)	106 (11)
Waist-hip ratio	0.83 (0.07)	0.85 (0.06)	0.86 (0.06)	0.87 (0.06)	0.88 (0.06)	0.89 (0.06)	0.89 (0.06)	0.87 (0.06)
Waist-height ratio	0.51 (0.05)	0.54 (0.04)	0.57 (0.04)	0.60 (0.04)	0.64 (0.05)	0.68 (0.05)	0.74 (0.07)	0.61 (0.08)
SBP, mmHg	118 (15)	120 (15)	122 (15)	124 (15)	127 (16)	128 (15)	132 (16)	124 (16)
DBP, mmHg	78 (10)	79 (10)	80 (10)	82 (10)	83 (10)	84 (10)	86 (10)	82 (10)
**Glycated haemoglobin**								
Mean (SD), %	5.3 (0.3)	5.3 (0.3)	5.4 (0.4)	5.5 (0.4)	5.5 (0.4)	5.6 (0.4)	5.7 (0.4)	5.5 (0.4)
**Long term medication use**								
Any anti-hypertensive	469 (6%)	594 (8%)	1427 (9%)	1843 (12%)	2244 (15%)	1379 (18%)	1744 (23%)	9700 (13%)
Any anti-thrombotic	157 (2%)	194 (3%)	325 (2%)	349 (2%)	440 (3%)	202 (3%)	272 (4%)	1939 (3%)
Any lipid lowering	24 (<0.5%)	44 (1%)	71 (<0.5%)	62 (<0.5%)	53 (<0.5%)	36 (<0.5%)	24 (<0.5%)	314 (<0.5%)

Conventions and exclusions as per Table 1. * Groupings of BMI are the top and bottom two-tenths and middle three-fifths of the sex-specific baseline distribution.

Webtable 3: Correlation of adiposity markers in 113,163 participants aged 35-74 at recruitment

	Waist circumference	Hip circumference	Waist-hip ratio	Waist-height ratio	
Hip circumference	0.80				Men
	0.81				Women
	**0.81**				**All**
Waist-hip ratio	0.69	0.13			Men
	0.56	-0.01			Women
	**0.60**	**0.02**			**All**
Waist-height ratio	0.92	0.69	0.70		Men
	0.95	0.74	0.58		Women
	**0.94**	**0.73**	**0.61**		**All**
BMI	0.82	0.76	0.45	0.85	Men
	0.83	0.84	0.23	0.84	Women
	**0.83**	**0.82**	**0.29**	**0.84**	**All**

BMI=Body Mass Index. The (Pearson) correlation coefficients shown for men and women are age-adjusted; those for all individuals combined are adjusted for age and sex.

Webtable 4: Relevance of markers of adiposity to cause-specific vascular-metabolic mortality at ages 40-74 years, before and after mutual adjustment for other adiposity markers - sensitivity analyses

		Death RR (95% CI) per 1SD higher usual level							
		Waist circumference		Waist-hip ratio		Waist-height ratio		Hip circumference	
Cause of death	No. of deaths	With basic adjustments	+hip-c +BMI	With basic adjustments	+BMI	With basic adjustments	+hip-c +weight	With basic adjustments	+waist-c +BMI
**(a) After additional adjustment for fruit and vegetable consumption, fried food intake and sleep***									
VASCULAR									
Cardiac	641	1.25 (1.15, 1.35)	1.28 (1.14, 1.44)	1.22 (1.11, 1.35)	1.16 (1.05, 1.29)	1.27 (1.18, 1.38)	1.32 (1.20, 1.45)	1.13 (1.05, 1.22)	0.80 (0.72, 0.89)
Stroke	215	1.28 (1.11, 1.48)	1.45 (1.18, 1.77)	1.28 (1.08, 1.52)	1.23 (1.03, 1.46)	1.42 (1.24, 1.64)	1.77 (1.50, 2.09)	1.11 (0.97, 1.27)	0.78 (0.65, 0.94)
Other vascular	83	1.49 (1.19, 1.87)	1.54 (1.10, 2.16)	1.34 (1.01, 1.77)	1.27 (0.95, 1.69)	1.54 (1.26, 1.90)	1.47 (1.13, 1.91)	1.38 (1.15, 1.67)	1.14 (0.85, 1.52)
Subtotal: Any vascular	939	1.28 (1.19, 1.37)	1.34 (1.22, 1.48)	1.25 (1.15, 1.35)	1.19 (1.09, 1.29)	1.33 (1.25, 1.42)	1.43 (1.32, 1.54)	1.15 (1.08, 1.22)	0.82 (0.75, 0.90)
METABOLIC									
Renal/Acute diabetic crisis	254	1.57 (1.38, 1.80)	1.56 (1.29, 1.88)	1.36 (1.16, 1.59)	1.20 (1.03, 1.41)	1.58 (1.38, 1.80)	1.55 (1.32, 1.81)	1.37 (1.22, 1.54)	0.85 (0.73, 1.01)
Hepatobiliary	387	1.30 (1.17, 1.45)	1.58 (1.36, 1.84)	1.39 (1.23, 1.57)	1.33 (1.17, 1.52)	1.33 (1.21, 1.48)	1.53 (1.35, 1.74)	1.12 (1.02, 1.23)	0.72 (0.63, 0.82)
Subtotal: Any metabolic	641	1.41 (1.30, 1.53)	1.57 (1.40, 1.77)	1.38 (1.25, 1.52)	1.28 (1.16, 1.41)	1.42 (1.31, 1.54)	1.54 (1.39, 1.69)	1.22 (1.14, 1.31)	0.77 (0.70, 0.86)
ALL VASCULAR-METABOLIC	1580	1.33 (1.26, 1.40)	1.43 (1.33, 1.54)	1.30 (1.22, 1.38)	1.22 (1.15, 1.31)	1.37 (1.30, 1.44)	1.47 (1.39, 1.57)	1.18 (1.12, 1.23)	0.80 (0.75, 0.86)
**(b) After inclusion of participants with undiagnosed diabetes (no previously-diagnosed diabetes at recruitment, but glycosylated haemoglobin at least 6.5%)**									
VASCULAR									
Cardiac	667	1.26 (1.16, 1.36)	1.27 (1.13, 1.42)	1.25 (1.13, 1.38)	1.19 (1.07, 1.31)	1.29 (1.19, 1.39)	1.33 (1.21, 1.46)	1.14 (1.06, 1.23)	0.81 (0.73, 0.90)
Stroke	224	1.32 (1.15, 1.52)	1.47 (1.21, 1.80)	1.30 (1.10, 1.53)	1.23 (1.04, 1.45)	1.45 (1.27, 1.66)	1.81 (1.54, 2.14)	1.12 (0.99, 1.28)	0.73 (0.61, 0.88)
Other vascular	86	1.44 (1.15, 1.80)	1.39 (1.00, 1.93)	1.26 (0.96, 1.65)	1.19 (0.91, 1.57)	1.49 (1.22, 1.82)	1.36 (1.05, 1.77)	1.38 (1.15, 1.67)	1.20 (0.90, 1.59)
Subtotal: Any vascular	977	1.29 (1.21, 1.38)	1.33 (1.21, 1.46)	1.26 (1.16, 1.36)	1.20 (1.10, 1.30)	1.34 (1.26, 1.43)	1.43 (1.33, 1.55)	1.16 (1.09, 1.23)	0.82 (0.75, 0.89)
METABOLIC									
Renal/Acute diabetic crisis	269	1.62 (1.42, 1.84)	1.57 (1.31, 1.89)	1.39 (1.20, 1.62)	1.23 (1.05, 1.43)	1.59 (1.40, 1.81)	1.53 (1.31, 1.78)	1.39 (1.25, 1.55)	0.83 (0.71, 0.98)
Hepatobiliary	400	1.32 (1.19, 1.47)	1.58 (1.36, 1.84)	1.38 (1.22, 1.57)	1.32 (1.16, 1.49)	1.34 (1.21, 1.48)	1.51 (1.34, 1.71)	1.13 (1.03, 1.25)	0.72 (0.63, 0.82)
Subtotal: Any metabolic	669	1.44 (1.33, 1.56)	1.58 (1.41, 1.77)	1.39 (1.26, 1.53)	1.28 (1.16, 1.41)	1.43 (1.32, 1.55)	1.52 (1.38, 1.67)	1.24 (1.16, 1.33)	0.77 (0.69, 0.85)
ALL VASCULAR-METABOLIC	1646	1.35 (1.28, 1.42)	1.42 (1.32, 1.53)	1.31 (1.23, 1.39)	1.23 (1.15, 1.31)	1.38 (1.31, 1.45)	1.47 (1.38, 1.56)	1.19 (1.14, 1.25)	0.80 (0.74, 0.85)
**(c) After restricting to never smokers only**									
VASCULAR									
Cardiac	268	1.24 (1.10, 1.41)	1.34 (1.12, 1.59)	1.20 (1.02, 1.40)	1.16 (0.99, 1.37)	1.26 (1.12, 1.42)	1.32 (1.14, 1.53)	1.16 (1.03, 1.30)	0.88 (0.74, 1.03)
Stroke	98	1.47 (1.18, 1.82)	1.29 (0.96, 1.74)	1.27 (1.00, 1.61)	1.17 (0.92, 1.49)	1.54 (1.25, 1.90)	1.63 (1.27, 2.08)	1.28 (1.03, 1.59)	0.81 (0.61, 1.09)
Other vascular	36	2.02 (1.41, 2.89)	1.36 (0.81, 2.28)	1.51 (1.04, 2.19)	1.35 (0.93, 1.97)	2.04 (1.47, 2.81)	1.57 (1.04, 2.36)	1.81 (1.30, 2.53)	1.29 (0.78, 2.12)
Subtotal: Any vascular	402	1.36 (1.23, 1.51)	1.34 (1.16, 1.55)	1.25 (1.10, 1.41)	1.19 (1.05, 1.34)	1.40 (1.27, 1.54)	1.42 (1.26, 1.60)	1.25 (1.14, 1.38)	0.90 (0.79, 1.04)
METABOLIC									
Renal/Acute diabetic crisis	107	1.60 (1.32, 1.94)	1.36 (1.02, 1.81)	1.22 (0.96, 1.55)	1.11 (0.87, 1.41)	1.65 (1.35, 2.02)	1.38 (1.08, 1.77)	1.50 (1.27, 1.77)	1.01 (0.78, 1.31)
Hepatobiliary	143	1.41 (1.19, 1.68)	1.32 (1.03, 1.70)	1.27 (1.03, 1.56)	1.18 (0.96, 1.46)	1.45 (1.22, 1.71)	1.49 (1.21, 1.84)	1.27 (1.09, 1.49)	0.74 (0.59, 0.94)
Subtotal: Any metabolic	250	1.51 (1.32, 1.71)	1.35 (1.13, 1.60)	1.25 (1.07, 1.46)	1.39 (1.14, 1.68)	1.53 (1.34, 1.74)	1.33 (1.13, 1.56)	1.38 (1.23, 1.54)	1.32 (1.13, 1.54)
ALL VASCULAR-METABOLIC	652	1.42 (1.31, 1.54)	1.34 (1.20, 1.50)	1.25 (1.13, 1.37)	1.17 (1.06, 1.29)	1.45 (1.34, 1.57)	1.44 (1.31, 1.58)	1.30 (1.21, 1.40)	0.88 (0.79, 0.98)

Webtable 4: Relevance of markers of adiposity to cause-specific vascular-metabolic mortality at ages 40-74 years, before and after mutual adjustment for other adiposity markers - sensitivity analyses

		Death RR (95% CI) per 1SD higher usual level							
		Waist circumference		Waist-hip ratio		Waist-height ratio		Hip circumference	
Cause of death	No. of deaths	With basic adjustments	+hip-c +BMI	With basic adjustments	+BMI	With basic adjustments	+hip-c +weight	With basic adjustments	+waist-c +BMI
**(d) Without correction for regression dilution bias****									
VASCULAR									
Cardiac	641	1.22 (1.14, 1.32)	1.26 (1.13, 1.40)	1.12 (1.05, 1.20)	0.81 (0.73, 0.89)	1.19 (1.09, 1.30)	1.14 (1.05, 1.24)	1.26 (1.17, 1.35)	1.30 (1.19, 1.42)
Stroke	215	1.26 (1.10, 1.44)	1.41 (1.17, 1.70)	1.11 (0.98, 1.25)	0.79 (0.66, 0.94)	1.24 (1.07, 1.44)	1.20 (1.03, 1.39)	1.39 (1.22, 1.59)	1.71 (1.46, 2.00)
Other vascular	84	1.43 (1.17, 1.76)	1.48 (1.09, 2.01)	1.36 (1.14, 1.63)	1.15 (0.88, 1.51)	1.27 (1.00, 1.61)	1.22 (0.95, 1.56)	1.49 (1.23, 1.80)	1.40 (1.10, 1.80)
Subtotal: Any vascular	940	1.25 (1.18, 1.33)	1.31 (1.20, 1.43)	1.14 (1.08, 1.21)	0.83 (0.76, 0.90)	1.21 (1.13, 1.30)	1.16 (1.08, 1.25)	1.31 (1.23, 1.39)	1.40 (1.30, 1.50)
METABOLIC									
Renal/Acute diabetic crisis	256	1.53 (1.36, 1.73)	1.51 (1.27, 1.79)	1.36 (1.23, 1.52)	0.86 (0.74, 1.00)	1.32 (1.15, 1.51)	1.18 (1.03, 1.36)	1.55 (1.37, 1.76)	1.52 (1.31, 1.76)
Hepatobiliary	389	1.29 (1.17, 1.42)	1.54 (1.34, 1.77)	1.12 (1.02, 1.23)	0.73 (0.64, 0.83)	1.34 (1.20, 1.49)	1.29 (1.15, 1.44)	1.33 (1.20, 1.46)	1.51 (1.34, 1.69)
Subtotal: Any metabolic	645	1.39 (1.29, 1.49)	1.53 (1.37, 1.70)	1.22 (1.14, 1.31)	0.78 (0.71, 0.86)	1.33 (1.22, 1.45)	1.24 (1.14, 1.36)	1.41 (1.31, 1.52)	1.51 (1.38, 1.65)
ALL VASCULAR-METABOLIC	1585	1.30 (1.24, 1.37)	1.40 (1.30, 1.49)	1.17 (1.12, 1.23)	0.81 (0.76, 0.86)	1.26 (1.19, 1.33)	1.19 (1.13, 1.26)	1.35 (1.29, 1.41)	1.44 (1.36, 1.53)

Analyses, conventions and main exclusion criteria as per Table 2. Analyses presented in this table further include or exclude participants, or have other adjustments, as described. *Data present for additional covariates (fruit and vegetable consumption (days per week eaten: none, 1-2, 3-4, 5-7), fried food intake (days per week eaten: none, 1-2, 3-4, 5-7) and sleep (integer number of hours slept per night)) in 99.9% participants. **One baseline SD higher level of the adiposity markers was 10.8 cm for waist circumference, 9.3 cm for hip circumference, 0.061 for waist-hip ratio and 0.070 for waist-height ratio.

Webtable 5: Relevance of markers of adiposity to cause-specific non-vascular-metabolic mortality at ages 40-74 years, before and after mutual adjustment for other adiposity markers

		Death RR (95% CI) per 1SD higher usual level							
		Waist circumference		Waist-hip ratio		Waist-height ratio		Hip circumference	
**Cause of death**	**No. of deaths**	**With basic adjustments**	**+hip-c +BMI**	**With basic adjustments**	**+BMI**	**With basic adjustments**	**+hip-c +weight**	**With basic adjustments**	**+waist-c +BMI**
NON-VASCULAR-METABOLIC									
Neoplastic	924	1.13 (1.05, 1.20)	1.14 (1.03, 1.25)	1.10 (1.02, 1.19)	1.09 (1.01, 1.18)	1.08 (1.01, 1.16)	0.99 (0.91, 1.07)	1.10 (1.03, 1.17)	1.02 (0.93, 1.12)
Respiratory	371	1.33 (1.21, 1.47)	1.64 (1.41, 1.91)	1.33 (1.17, 1.50)	1.31 (1.16, 1.49)	1.42 (1.28, 1.56)	1.60 (1.41, 1.82)	1.18 (1.08, 1.29)	0.80 (0.70, 0.92)
Infective	223	1.38 (1.21, 1.59)	1.73 (1.42, 2.11)	1.41 (1.21, 1.66)	1.39 (1.18, 1.63)	1.41 (1.23, 1.62)	1.59 (1.34, 1.88)	1.22 (1.08, 1.37)	0.85 (0.71, 1.02)
Other/Ill-defined/External	299	1.02 (0.90, 1.14)	1.14 (0.96, 1.35)	1.00 (0.86, 1.16)	1.03 (0.89, 1.20)	1.03 (0.92, 1.16)	1.16 (1.01, 1.33)	1.00 (0.90, 1.11)	0.92 (0.79, 1.08)
Any non-vascular-metabolic	1817	1.18 (1.12, 1.23)	1.28 (1.20, 1.38)	1.16 (1.10, 1.23)	1.16 (1.09, 1.22)	1.18 (1.12, 1.23)	1.19 (1.12, 1.26)	1.11 (1.06, 1.16)	0.94 (0.88, 1.00)

Analyses, conventions and exclusion criteria as per Table 2.
